# A heterobimetallic tetrahedron from a linear platinum(II)-bis(acetylide) metalloligand

**DOI:** 10.3762/bjoc.16.220

**Published:** 2020-11-03

**Authors:** Matthias Hardy, Marianne Engeser, Arne Lützen

**Affiliations:** 1 University of Bonn, Kekulé-Institute of Organic Chemistry and Biochemistry, Gerhard-Domagk-Str. 1, D-53121 Bonn, Germany

**Keywords:** cage compounds, heterobimetallic complexes, pyridylimine ligands, self-assembly, supramolecular chemistry

## Abstract

Employing 4-ethynylaniline as a simple organic ligand we were able to prepare the stable *trans*-bis(acetylide)platinum(II) complex [Pt(L^1^)_2_(PBu_3_)_2_] as a linear metalloligand. The reaction of this metalloligand with iron(II) cations and pyridine-2-carbaldehyde according to the subcomponent self-assembly approach yielded decanuclear heterobimetallic tetrahedron [Fe_4_Pt_6_(L^2^)_12_](OTf)_8_. Thus, combination of these two design concepts – the subcomponent self-assembly strategy and the complex-as-a-ligand approach – ensured a fast and easy synthesis of large heterobimetallic coordination cages of tetrahedral shape with a diameter of more than 3 nm as a mixture of all three possible *T*-, *S*_4_- and *C*_3_-symmetric diastereomers. The new complexes were characterized by NMR and UV–vis spectroscopy and ESI mass spectrometry. Using GFN2-xTB we generated energy-minimized models of the diastereomers of this cage that further corroborated the results from analytical findings.

## Introduction

The understanding of the general design principles for the self-assembly of metallosupramolecular aggregates [1–5] allowed to access more and more complex and large assemblies over the past decades like sophisticated cage-in-ring structures [6–7], interlocked rings [8] and catenanes [9–12] or giant spheres [13–14].

Increasing the structural complexity, however, usually implies growing synthetic efforts that have to be made to obtain the necessary organic ligands [15–17]. A great way to simplify ligand syntheses is the subcomponent self-assembly strategy [18–23]. Following this strategy, the actual ligand is generated in situ during the self-assembly process via reversible formation of covalent bonds, in most cases imine bonds. Despite this achievement, addressing even higher complexity with homometallic assemblies becomes increasingly difficult to achieve. An approach to tackle these limitations is the use of not only one, but two different types of metal cations to form heterobimetallic aggregates [24–26].

Introducing two different types of metal cations within one discrete aggregate offers the chance to combine the symmetry elements of both metal centers, and hence, access new geometries. Reliable design strategies like the complex-as-a-ligand approach made the assembly of heterobimetallic structures predictable; hence, there is a rapidly growing number of heterobimetallic structures that has recently been described in the literature, such as, e.g., helicates [27–28], cubes [29–31], trigonal bipyramids [21,32–33], boxes [34–36], prismatic cages [37], or some truly unique other shapes [38–39]. At the same time, heterobimetallic cages not only offer the chance to obtain new geometries, the combination of two different metal cations within one aggregate might also lead to enhanced or even entirely new properties and functions [40]. Searching the literature, however, it is striking that the number of heterobimetallic tetrahedra [41–42] is noticeably small, compared to homometallic examples. Thus, we were wondering if we can combine the complex-as-a-ligand and the subcomponent self-assembly approach to access such a heterobimetallic complex of tetrahedral shape.

## Results and Discussion

Whereas homometallic metallosupramolecular tetrahedra can readily be assembled from linear divalent or planar trivalent ligands with octahedrally coordinating metal cations, the search for subtle building blocks is obviously more difficult, if the organoligand should be replaced by a metalloligand with a similar geometry. Numerous examples in the literature prove platinum(II) ions to be especially suitable to prepare a linear metalloligand due to their highly predictable square-planar coordination environment that can easily be employed to access tetravalent planar or divalent V-shaped *cis*- or linear *trans*-configurated complexes [43–45].

In this context platinum(II)-bis(acetylide) complexes [46–47] proved useful as building blocks for the construction of polymers [48], rings [49–50] and cages [36,42,51]. These neutral compounds are usually easy-to-access, rather stable and the *cis-* and *trans-*isomers can be separated efficiently.

**Scheme 1:**
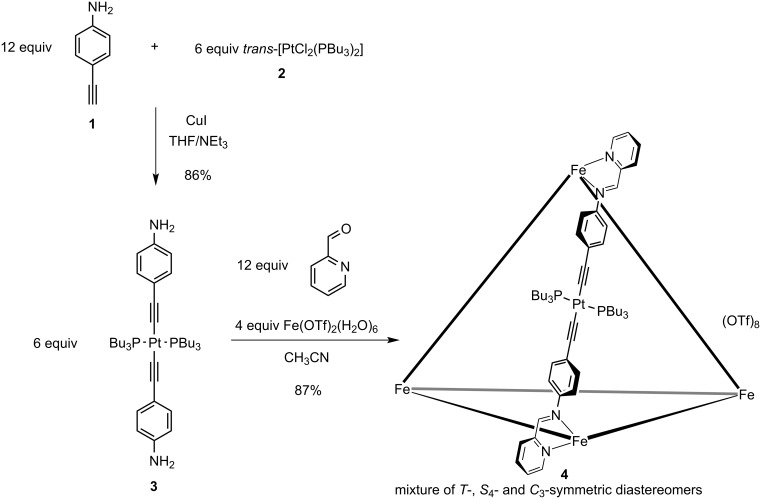
Stepwise assembly of the heterobimetallic tetrahedron **4**, starting from 4-ethynylaniline (**1**) and *trans*-[Pt(PBu_3_)_2_Cl_2_] (**2**) that were combined to yield the linear metalloligand **3** that was subsequently used to assemble the desired decanuclear heterobimetallic aggregate upon treatment with pyridine-2-carbaldehyde and iron(II) triflate hexahydrate. Please note that not all organic ligands in cage **4** are shown, however, each iron(II) cation is coordinated by three pyridylimine chelates.

In this work we employed the linear platinum(II)-bis(acetylide) metalloligand **3** that bears two 4-aniline moieties (Scheme 1). In order to synthesize this key compound, we followed known procedures to first isolate 4-ethynylaniline (**1**) [52] that was subsequently transformed into metalloligand **3** [53] upon treatment with *trans*-[Pt(PBu_3_)_2_Cl_2_] (**2**). The aniline moieties in **3** were further transformed into chelating pyridylimine binding sites in the following subcomponent self-assembly process when six equivalents of metalloligand **3** were reacted with twelve equivalents of pyridine-2-carbaldehyde and four equivalents of iron(II) triflate hexahydrate in acetonitrile giving rise to the desired heterobimetallic [Fe_4_Pt_6_] complex as a mixture of diastereomers (Scheme 1). Despite the introduced imine functionalities, which are prone to hydrolysis, we did not observe any hydrolysis under the reaction conditions with at least 3 equivalents of water per imine function that originate from the hydrate salt and imine condensations.

Following this approach, the heterobimetallic cage **4** was obtained as a dark purple solid in 87% yield. Interestingly, complex **4** turned out to be rather stable as a solid under ambient conditions for several weeks, while metalloligand **3** needs to be stored in an argon atmosphere at 3 °C and **1** even needs to be stored only in an argon atmosphere at −18 °C to prevent decomposition. The reduced stability of the amine precursors compared to cage **4** might be a result from the higher electron-donating capability of amine functions and thus a higher tendency to undergo one-electron oxidation reactions when stored under ambient conditions.

**Figure 1:**
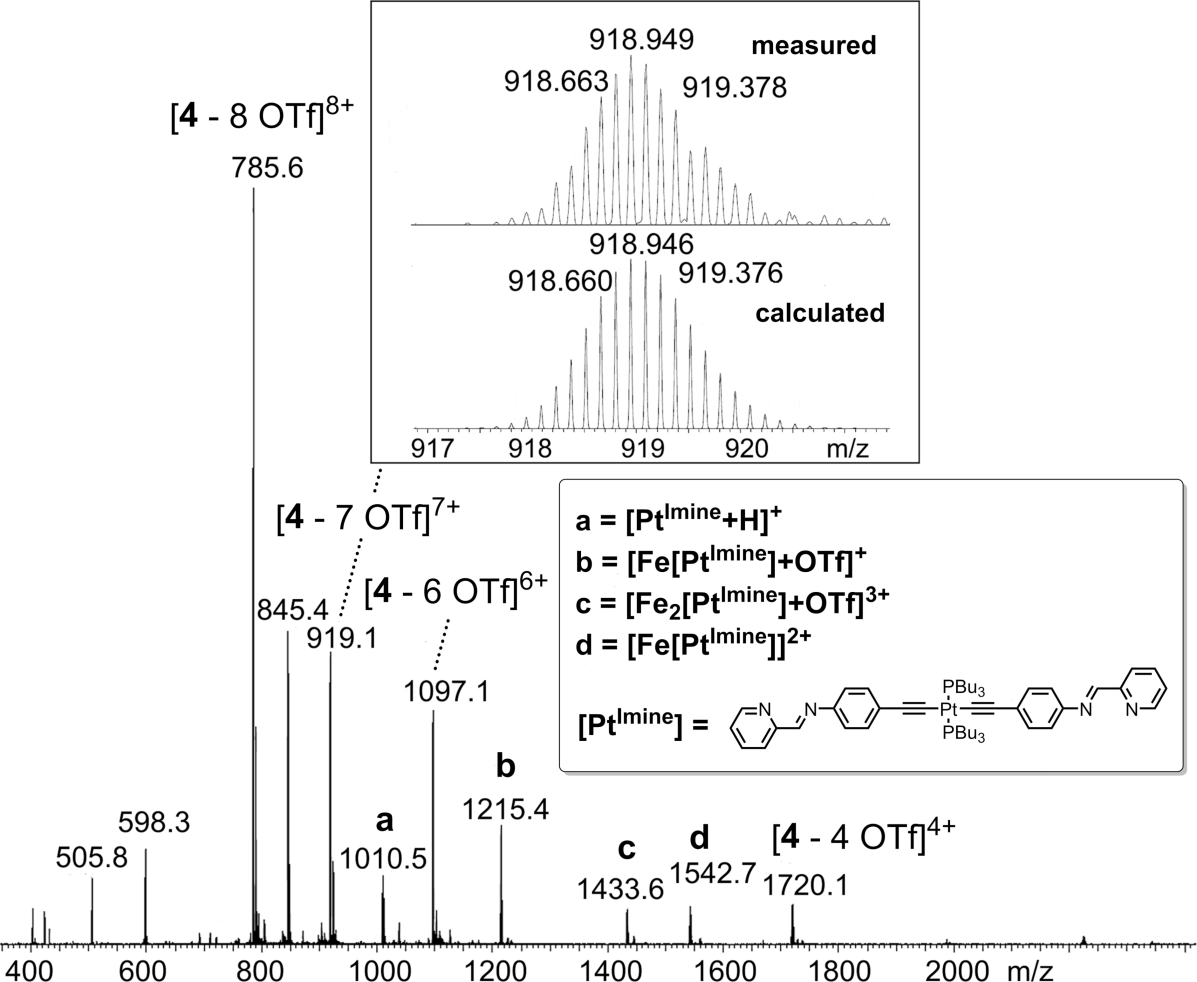
ESI(+) mass spectrum of heterobimetallic complex **4**. The top inset shows the experimentally observed and the calculated isotopic pattern for the signal of [**4** − 7 OTf]^7+^. The lower inset shows observed cage fragments a–d.

In order to check the composition of heterobimetallic **4** we performed mass spectrometric experiments first. Figure 1 shows the ESI mass spectrum of metallosupramolecular cage **4**, showing a series of signals with different charge states that could be assigned to **4**. The mass spectrum also shows that the cage easily fragments upon ESI, as additional signals a–d were detected.

**Figure 2:**
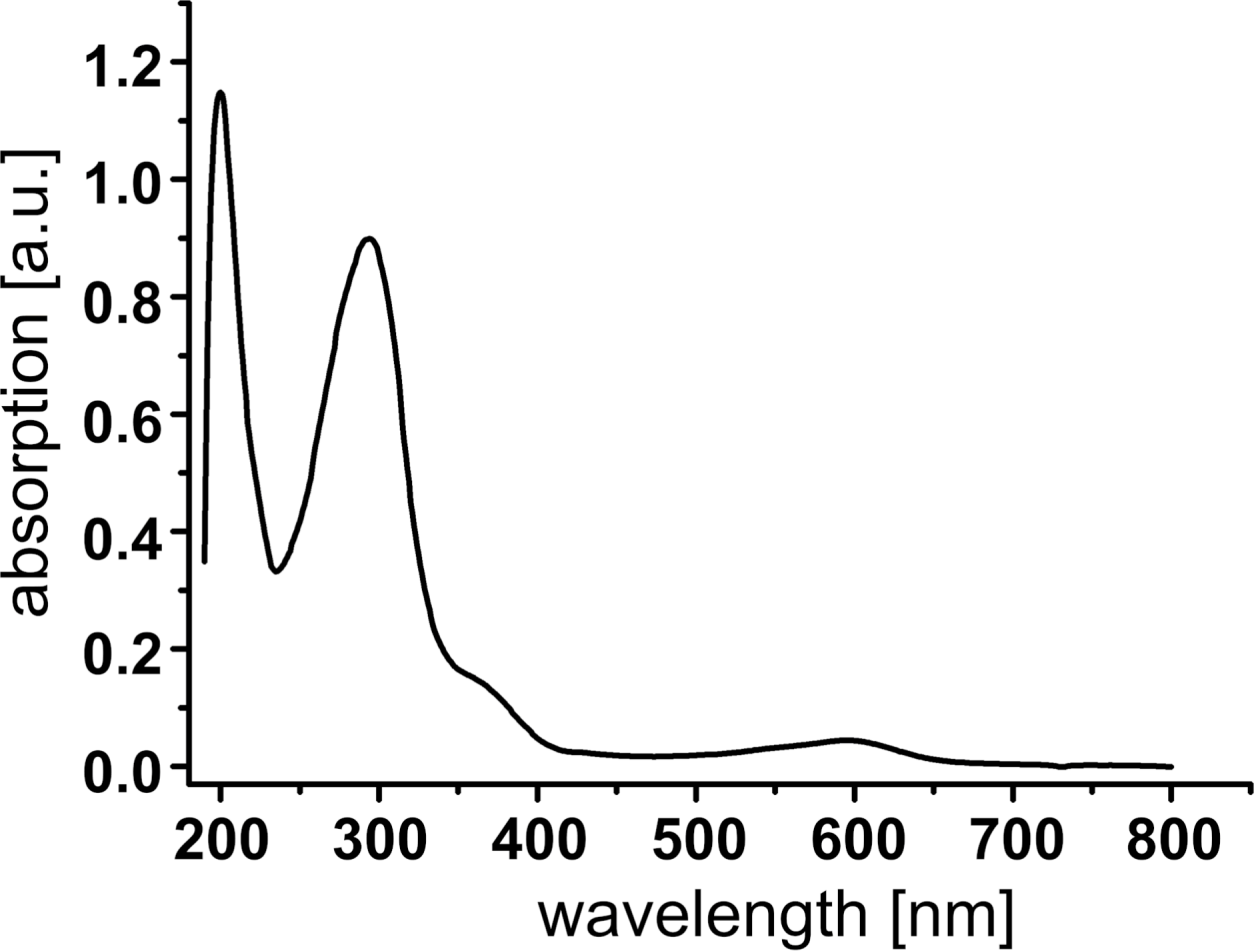
UV–vis spectrum of heterobimetallic complex **4** (1150 µM in acetonitrile at 295 K, 0.01 mm cuvette).

The successful formation of iron(II)-containing metallosupramolecular tetrahedron **4** could also be proven by UV–vis spectroscopy. The spectrum of tetrahedron **4** in acetonitrile solution revealed multiple absorption maxima. The most prominent maxima are located at 200 and 294 nm with a shoulder at 360 nm, probably corresponding to π–π* transitions from the aromatic systems and the triple bonds. Finally, a less intense maximum at 595 nm can be assigned to iron-centered transitions, which are typical for low-spin iron(II) complexes in tris(pyridylimine) binding pockets [54] (Figure 2).

**Figure 3:**
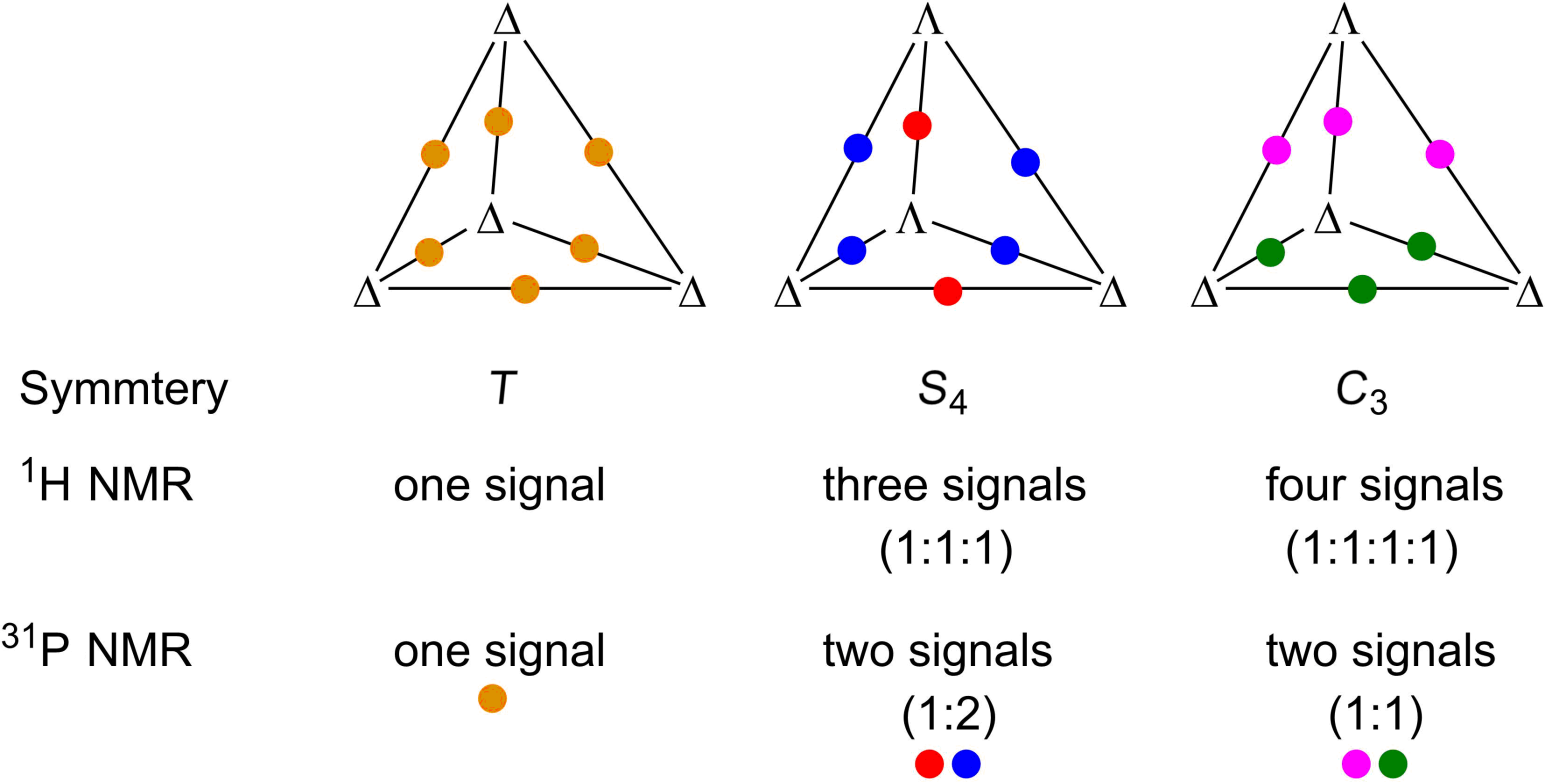
Schematic representation of symmetry-considerations concerning possible diastereomeric tetrahedra. The figure summarizes the cage symmetries and the expected observations in the ^1^H and ^31^P NMR spectra, looking at one chemically equivalent position. The colored dots represent chemically and magnetically equivalent positions of P atoms.

Also, the NMR spectroscopic analysis revealed the successful formation of discrete complexes that contain four newly formed stereogenic iron(II) centers. However, self-assembly of a metallosupramolecular tetrahedron with four stereogenic, octahedrally coordinated iron cations can give rise to three different diastereomers that adopt either *T-*, *S*_4_-, or *C*_3_-symmetry, respectively. Analysis of the ^1^H NMR spectrum of **4** (see Figure S1 in Supporting Information File 1 and also Figure 5 below) shows signals that clearly indicate the presence of various diastereomeric cages, and thus, the self-assembly does not occur in a diastereoselective manner. Unfortunately, the superposition of these signals made it impossible to clearly differentiate between these diastereomers. However, looking at the expected signal integrals with different configurations of the stereogenic metal centers (Figure 3) it is clear that all three possible diastereomers must be present in solution, as the observed signals cannot be explained with only one or two of these diastereomers.

**Figure 4:**
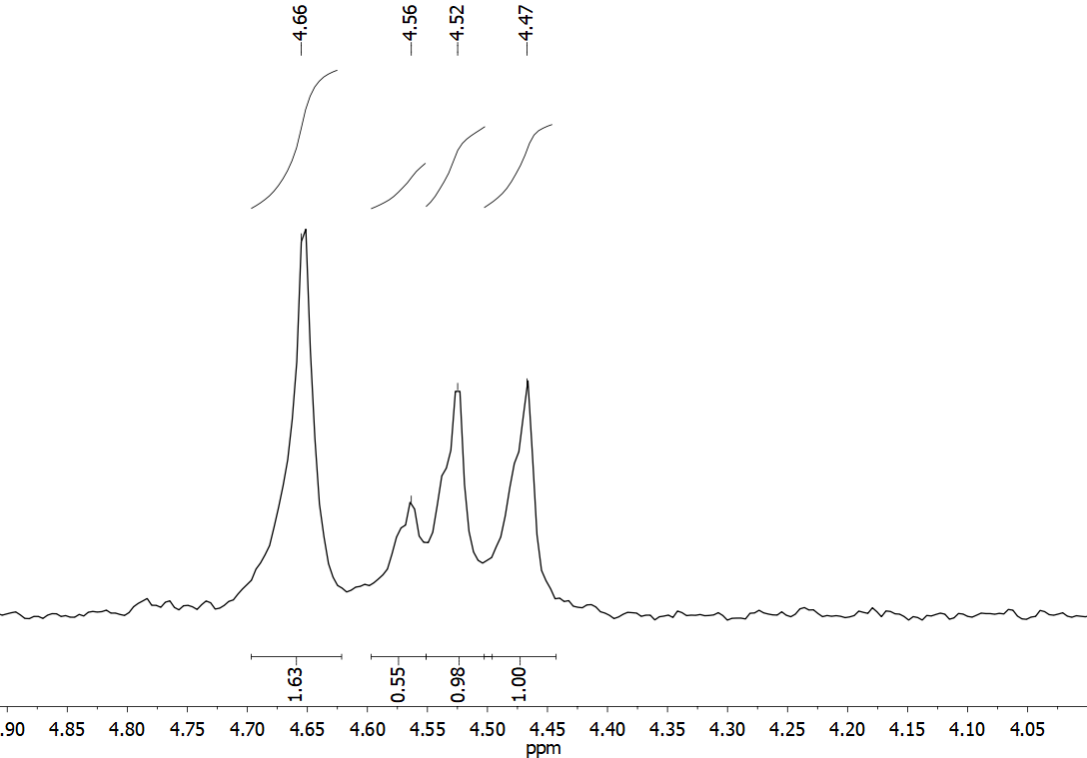
Detailed excerpt of the ^31^P NMR spectrum of **4** (202 MHz, acetonitrile-*d*_3_, 298 K).

This can also be seen when looking at the ^31^P NMR spectrum of **4** (see Figure 4 and Figure S4 in Supporting Information File 1).

Figure 4 shows that four signals are observed in the ^31^P NMR spectrum, which might be the case, if only *S*_4_ and *C*_3_ symmetric cages would be present. However, the integrals of these signals do not fit this assumption, and therefore, the only valid explanation is a mixture of all three diastereomers. A tentative assignment of the ^31^P NMR signals could be δ [ppm] = 4.47 (integral = 1, *C*_3_), 4.52 (integral = 1, *C*_3_), 4.56 (integral = 0.5, *S*_4_), 4.66 (integral = 1.6, *S*_4_ and *T*) but it must be noted that this assignment is not completely unambiguous as we were not able to detect the relative ratios of the diastereomeric cages with the necessary accuracy, since at least one of the signals is superposed.

**Figure 5:**
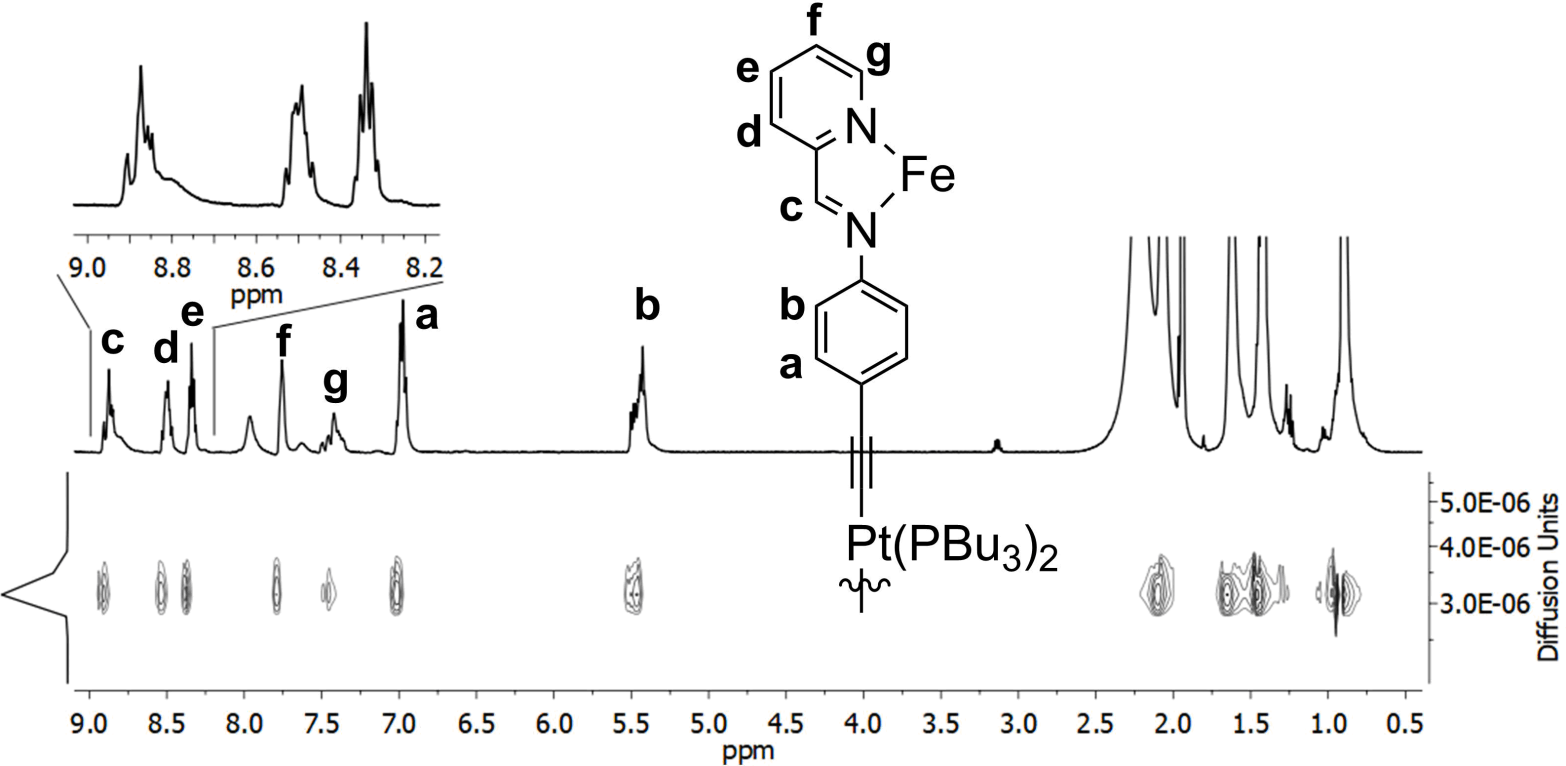
^1^H NMR and DOSY spectrum of heterobimetallic assembly **4** (500 MHz, acetonitilre-*d*_3_, 298 K).

Since all possible diastereomers hardly vary in size and shape, however, DOSY NMR experiments could nicely be used to prove that all of the detected signals correspond to species of similar size with a solvodynamic diameter of 33 Å (Figure 5) and do not belong to species of a different composition.

**Figure 6:**
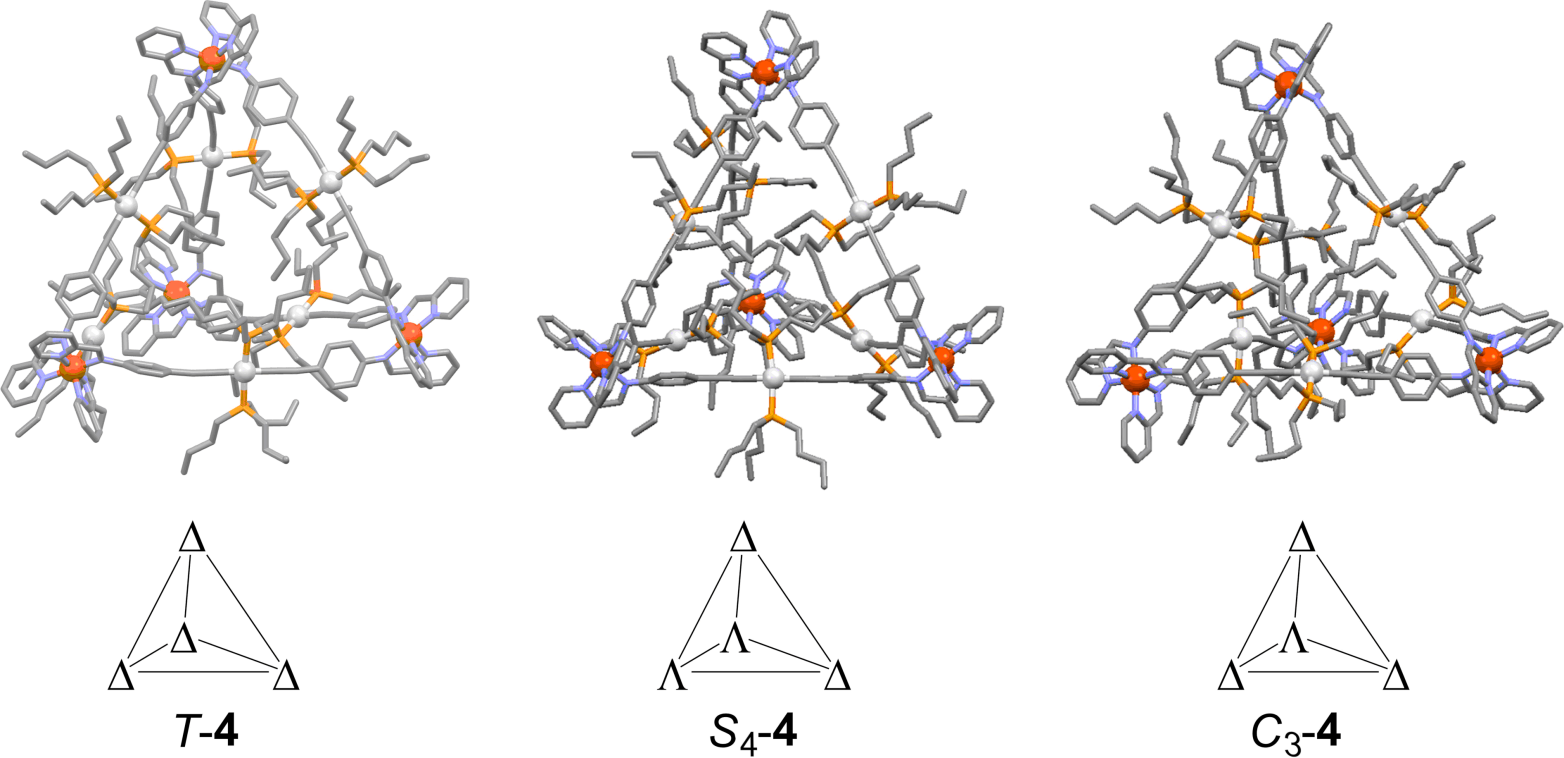
GFN2-xTB minimized gas phase models of the cationic units of all possible diastereomers of **4**. Color code: red – iron, white – platinum, orange – phosphorous, blue – nitrogen, grey – carbon. Hydrogen atoms are omitted for clarity. The stereogenic centers are defined in the schematic tetrahedra.

Unfortunately, despite our greatest efforts, we did not succeed to obtain single crystals of our metallosupramolecular assemblies that were suitable for single-crystal X-ray diffraction experiments. Therefore, we generated energy-minimized gas phase structures of **4**, employing a force-field approach using the GFN2-xTB approach recently established to model large (supra-)molecular entities with astonishing accuracy [55–56]. We minimized the structures of the cationic units of all possible diastereomers of cage **4** (Figure 6). From these models we could derive a theoretically expected diameter of *d*_calc_ = 31 Å that nicely corroborates the solvodynamic diameter from the DOSY experiment (*d*_h_ = 33 Å).

## Conclusion

In summary, we presented the self-assembly of a large decanuclear heterobimetallic tetrahedron that was readily obtained in a stepwise manner. The linear metalloligand **3** facilitated the formation of tetrahedral cages, when combined with pyridine-2-carbaldehyde and iron(II) cations in a subcomponent self-assembly approach. Unfortunately, the self-assembly process in solution did not occur in a diastereoselective manner as all possible diastereomers were observed. However, the DOSY and mass spectra clearly identified the tetrahedral cage **4** as the exclusive product. Ultimately, energy-minimized structures corroborated the results from the analytical spectra. Having established an easy access to these more than 3 nm-sized metallosupramolecular architectures with cavity volumes of approximately 140 Å^3^, subtle ligand modifications might be used to obtain analogues cages in future work to explore the properties of these system in terms of their host–guest chemistry or their magnetic behavior.

## Experimental

### General

All reagents and solvents were purchased from commercial sources and used as received without any further purification. NMR spectra were recorded on a Bruker Avance I 500 spectrometer. ^1^H NMR chemical shifts are reported relative to the residual solvent peak and ^13^C NMR chemical shifts are reported relative to the solvent peak. ^19^F and ^31^P NMR chemical shifts are reported relative to external references (CF_3_COOD in D_2_O for ^19^F and D_3_PO_4_ for ^31^P). In order to measure ^19^F and ^31^P NMR spectra, the NMR tube was equipped with a coaxial insert containing the external standards. ^1^H NMR data are reported as follows: chemical shift (δ) in ppm, multiplicity (dt = doublet of triplets, m = multiplet), coupling constant (*J*) in Hertz (Hz), integral, correlation of the proton. Low- and high-resolution electrospray ionization mass spectrometry (ESIMS) spectra were recorded on a Bruker Daltonic LTQ Orbitrap XL. The UV–vis spectrum was recorded on a Specord 200 spectrometer (Analytik Jena AG) at ambient temperature.

4-Ethynylaniline (**1**) [50], *trans*-[Pt(PBu_3_)_2_Cl_2_] (**2**) [57] and metalloligand **3** [53] were synthesized according to known literature protocols.


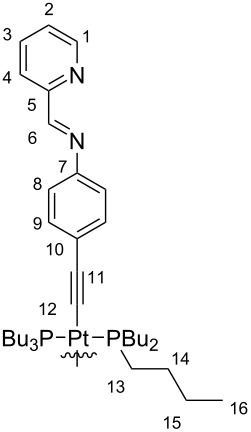


### Synthesis of the heterobimetallic tetrahedron **4**

*trans*-Bis(4-ethynylaniline)-bis(tributylphosphine)platinum(II) (7.00 mg, 8.41 µmol, 6.00 equiv) was dissolved in 1 mL of acetonitrile. Pyridine-2-carbaldehyde (1.80 mg, 16.8 µmol, 12.0 equiv) was added and the resulting solution was stirred for 30 minutes at room temperature. Then, iron(II) triflate hexahydrate (2.59 mg, 5.61 µmol, 4.00 equiv) was added and the solution was degassed by applying a vacuum and flushing with argon three times. The dark purple solution was stirred in an argon atmosphere for 16 hours at 50 °C. After cooling to room temperature, the solution was filtered and 3 mL of *n*-pentane were added to the filtrate. The resulting precipitate was collected, washed with *n*-pentane and diethyl ether and dried in a stream of air. The heterobimetallic cage **4** was obtained as a dark purple solid in 87% yield (9.12 mg, 1.22 µmol). ^1^H NMR (500 MHz, CD_3_CN, 298 K) δ [ppm] 8.91–8.85 (m, 12H, H-6), 8.51–8.48 (m, 12H, H-4), 8.36–8.32 (m, 12H, H-3), 7.76 (m, 12H, H-2), 7.42 (m, 12H, H-1), 7.02–6.94 (m, 12H, H-9), 5.50–5.41 (m, 24H, H-8), 2.09–2.04 (m, 72H, H-13), 1.64–1.60 (m, 72H, H-14), 1.45–1.40 (m, 72H, H-15), 0.90 (dt, *^3^J_16,15_* = 7.2 Hz, *^4^J_16,14_* = 2.3 Hz, 108H, H-16); ^13^C NMR (126 MHz, CD_3_CN, 298 K) δ [ppm] 174.0 (C-6), 156.3 (C-1), 148.8 (C-7, C-5), 140.3 (C-3), 132.0 (C-4), 131.4 (C-9), 130.6 (C-2), 122.2 (C-8), 108.8 (C-10), 26.2 (C-14), 24.4 (C-13, C-15), 14.2 (C-16) (Please note that the ^13^C NMR signals were assigned based on the analysis of the calibrated HSQC and HMBC spectra. Due to the complicated mixture of diastereomers the concentration was not sufficient to obtain a sufficiently resolved ^13^C NMR spectrum); ^31^P NMR (202 MHz, CD_3_CN, 298 K) δ [ppm] 4.66–4.47 (m); ^19^F NMR (470 MHz, CD_3_CN, 298 K) δ [ppm] −79.4 (C*F_3_*SO_3_^−^); DOSY (500 MHz, CD_3_CN, 298 K, τ = 150 ms): *D* = 3.33∙10^−10^ m^2^/s, *d*_h_ = 33 Å, *r*_h_ = 1.65 nm; ESI(+)-MS (CH_3_CN, M = {C_320_H_432_Fe_4_N_24_P_12_Pt_6_}^8+^) *m/z*: 1720.141 [M + 4OTf]^4+^, 1542.718 [Fe(C_52_H_72_N_4_P_2_Pt)_3_]^2+^, 1433.608 [Fe_2_(C_52_H_72_N_4_P_2_Pt) + OTf]^3+^, 1215.389 [Fe(C_52_H_72_N_4_P_2_Pt) + OTf]^+^, 1097.108 [M + 2OTf]^6+^, 1010.505 [C_52_H_72_N_4_P_2_Pt + H]^+^, 919.100 [M + OTf]^7+^, 785.594 [M]^8+^; HRMS (*m/z*): calcd for [C_320_H_432_Fe_4_N_24_P_12_Pt_6_+OTf]^7+^, 918.946; found, 918.949; UV–vis (CH_3_CN, 1150 µM, 0.01 mm cuvette) λ_max_ (nm): 200, 294, 360, 595.

## Supporting Information

File 1 Collection of the different NMR spectra recorded from heterobimetallic metallosupramolecular tetrahedron **4** and details regarding the energy-minimized structures of **4**.
