# Supplementary material for: A heterobimetallic tetrahedron from a linear platinum(II)-bis(acetylide) metalloligand
Source: Beilstein J Org Chem. 2020 Nov 3;16:2701–8. doi: 10.3762/bjoc.16.220 (PMC7653331; doi:10.3762/bjoc.16.220)
Supplement: File 1 — Collection of the different NMR spectra recorded from heterobimetallic metallosupramolecular tetrahedron 4 and details regarding the energy-minimized structures of 4. [file Beilstein_J_Org_Chem-16-2701-s001.pdf]

BEILSTEIN JOURNAL OF ORGANIC CHEMISTRY

# Supporting Information

for

# A heterobimetallic tetrahedron from a linear platinum(II)-bis(acetylide) metalloligand

Matthias Hardy, Marianne Engeser and Arne Lützen

*Beilstein J. Org. Chem.* **2020,** *16,* 2701–2708. doi:10.3762/bjoc.16.220

## Collection of the different NMR spectra recorded from heterobimetallic metallosupramolecular tetrahedron 4 and details regarding the energy-minimized structures of 4

License and Terms: This is a supporting information file under the terms of the Creative Commons Attribution License (https://creativecommons.org/licenses/by/4.0). Please note that the reuse, redistribution and reproduction in particular requires that the authors and source are credited.
The license is subject to the *Beilstein Journal of Organic Chemistry* terms and conditions: (https://www.beilstein-journals.org/bjoc)

## Table of Contents

NMR spectra of metallosupramolecular tetrahedron **4**.................................................................... S2

Selected isotopic pattern from the mass spectrum of metallosupramolecular tetrahedron **4**........ S8

Atom coordinates of energy-minimized cages **4**............................................................................ S9

## NMR spectra of metallosupramolecular tetrahedron 4

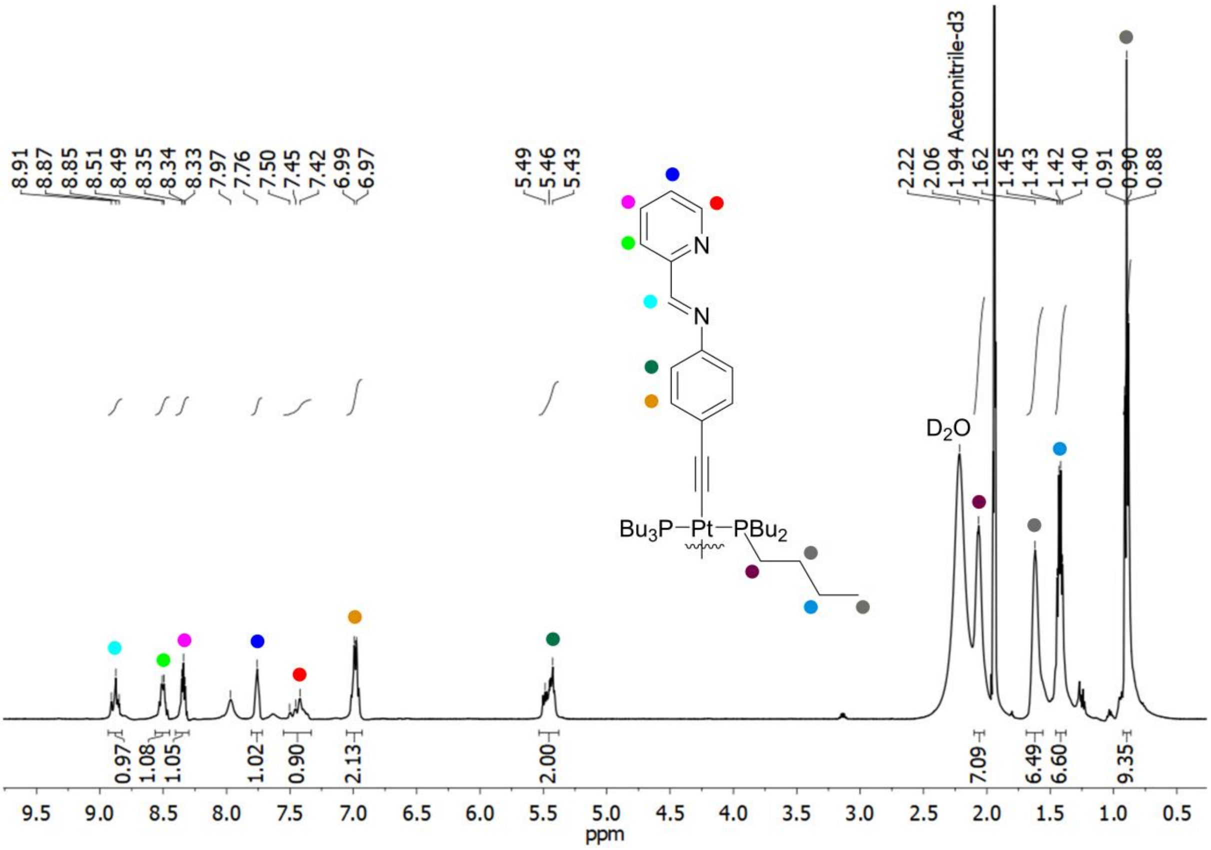


**Figure S1.** $^1$H NMR spectrum of **4** (500 MHz, acetonitrile-$d_3$, 298 K).

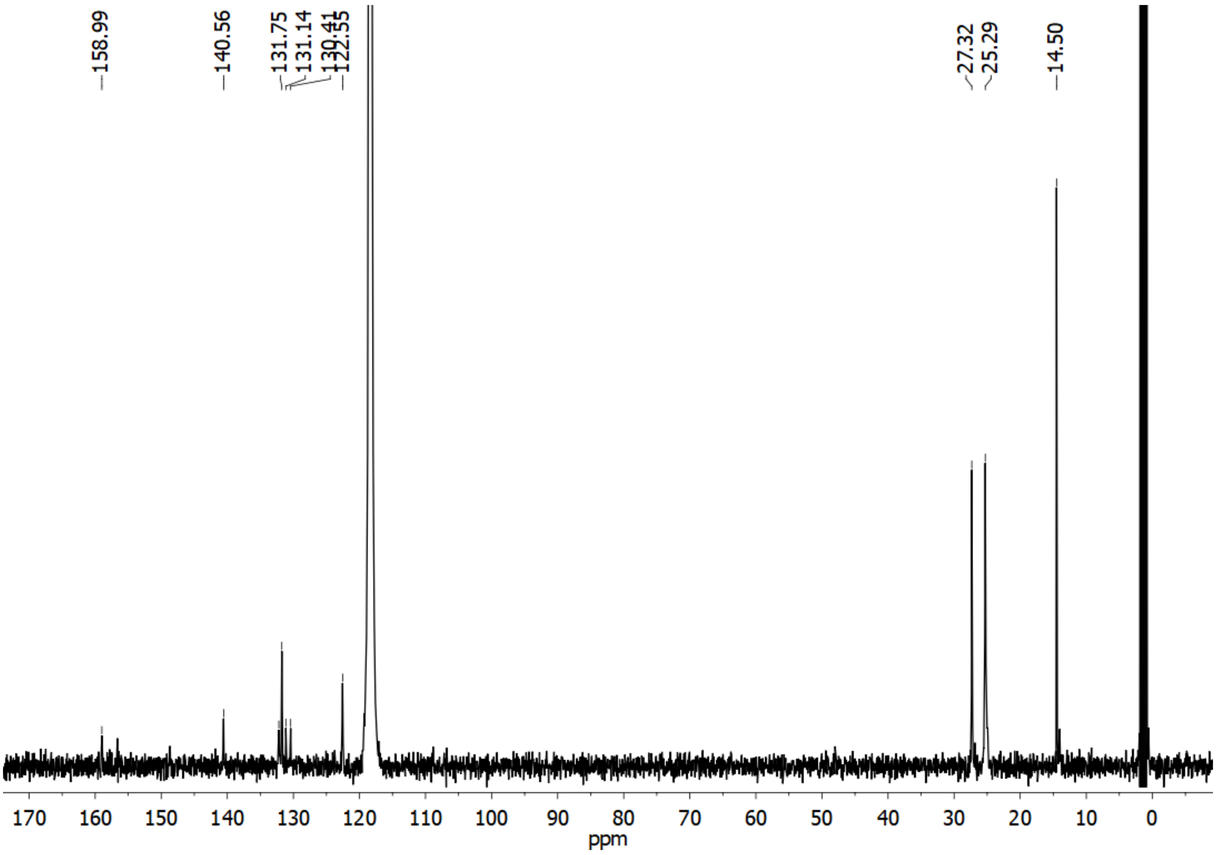


**Figure S2.** $^{13}C$ NMR spectrum of **4** (126 MHz, acetonitrile-*d*3, 298 K).

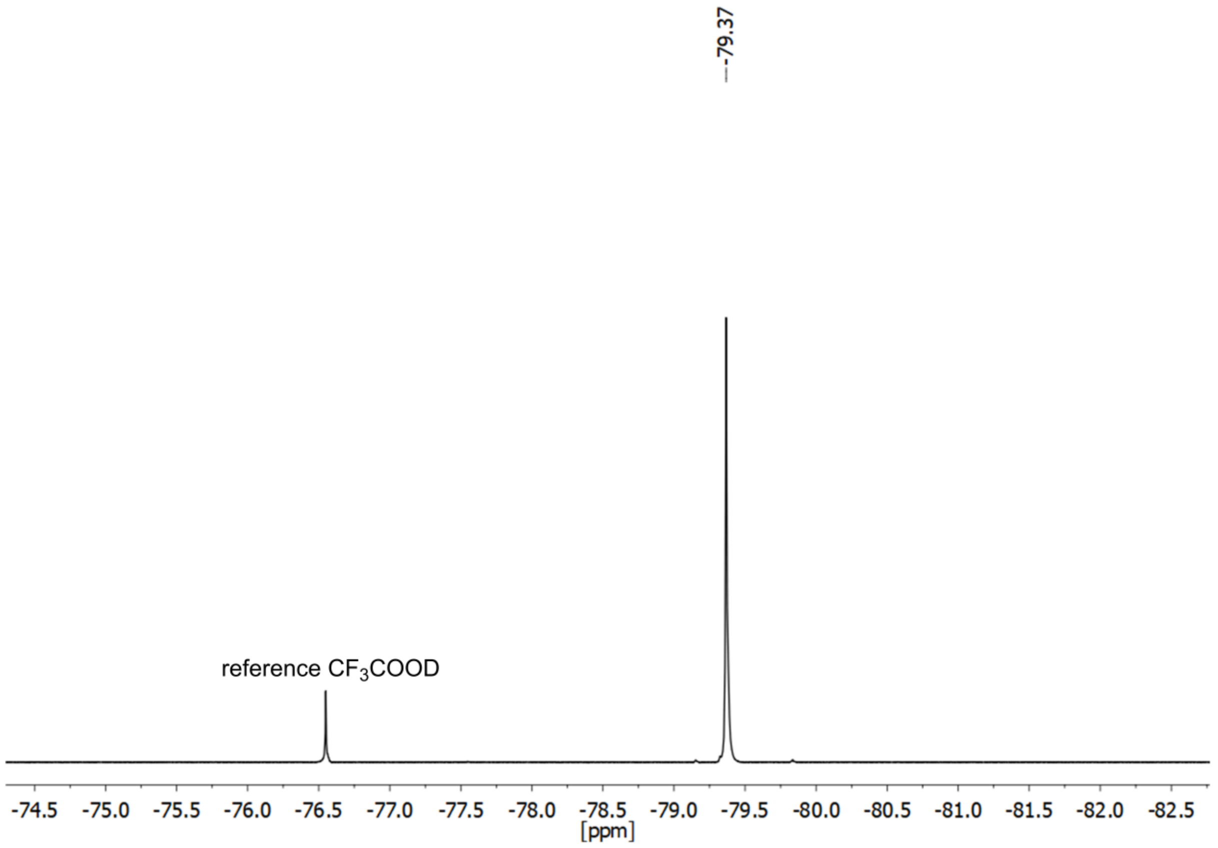


**Figure S3.** $^{19}F$ NMR spectrum of **4** (470 MHz, acetonitrile-*d*3, 298 K).

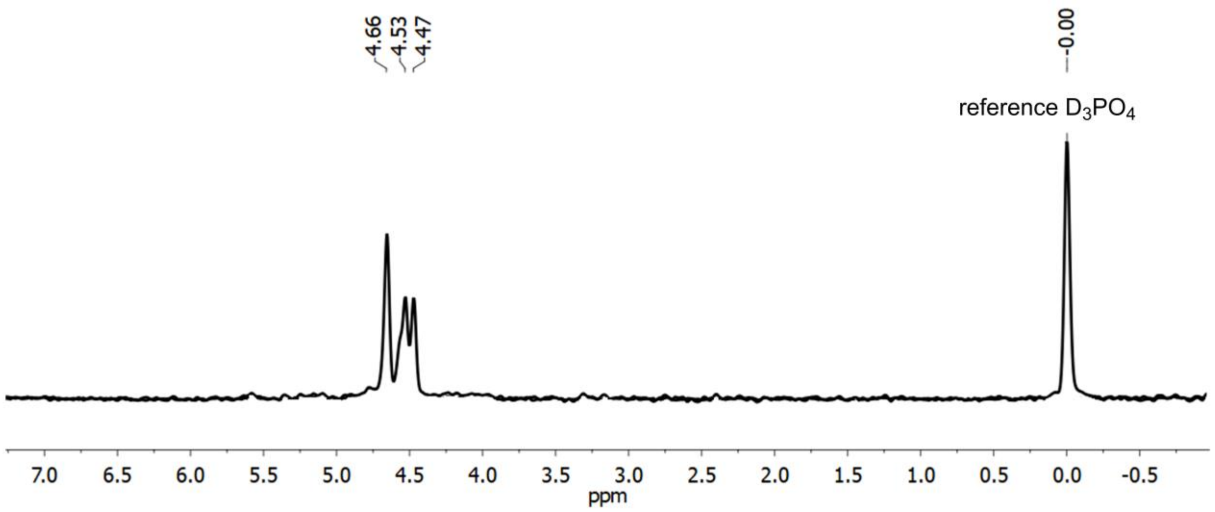


**Figure S4.** $^{31}$P NMR spectrum of **4** (202 MHz, acetonitrile-*d*3, 298 K).

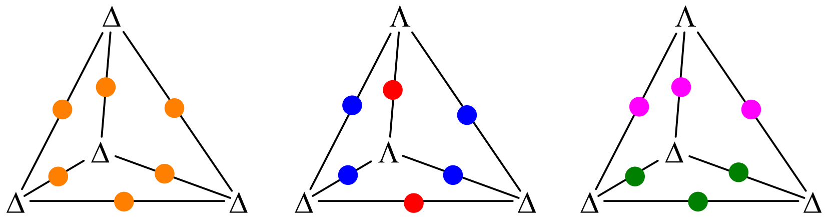


| Symmtery | T | $S_4$ | $C_3$ |
|---|---|---|---|
| $^{1}$H-NMR | one signal | three signals (1:1:1) | four signals (1:1:1:1) |
| $^{31}$P-NMR | one signal | two signals (1:2) | two signals (1:1) |

**Scheme S1.** Schematic representation of symmetry-considerations concerning possible diastereomeric tetrahedra. The table summarizes the cage symmetries and the expected observations in the $^{1}$H- and $^{31}$P-NMR spectra, looking at one chemically equivalent position. The coloured dots represent chemically and magnetically equivalent positions.

The $^{1}$H NMR spectrum of **4** shows signals that clearly indicate the presence of various diastereomeric cages, but unfortunately the superposition of these signals makes it impossible to differentiate between these diastereomers. However, looking at the expected signal integrals with different configurations of the stereogenic centers (Scheme S1) it is clear, that all three possible diastereomers must be present in solution, as the observed signals cannot be explained with only one or two of these diastereomers.

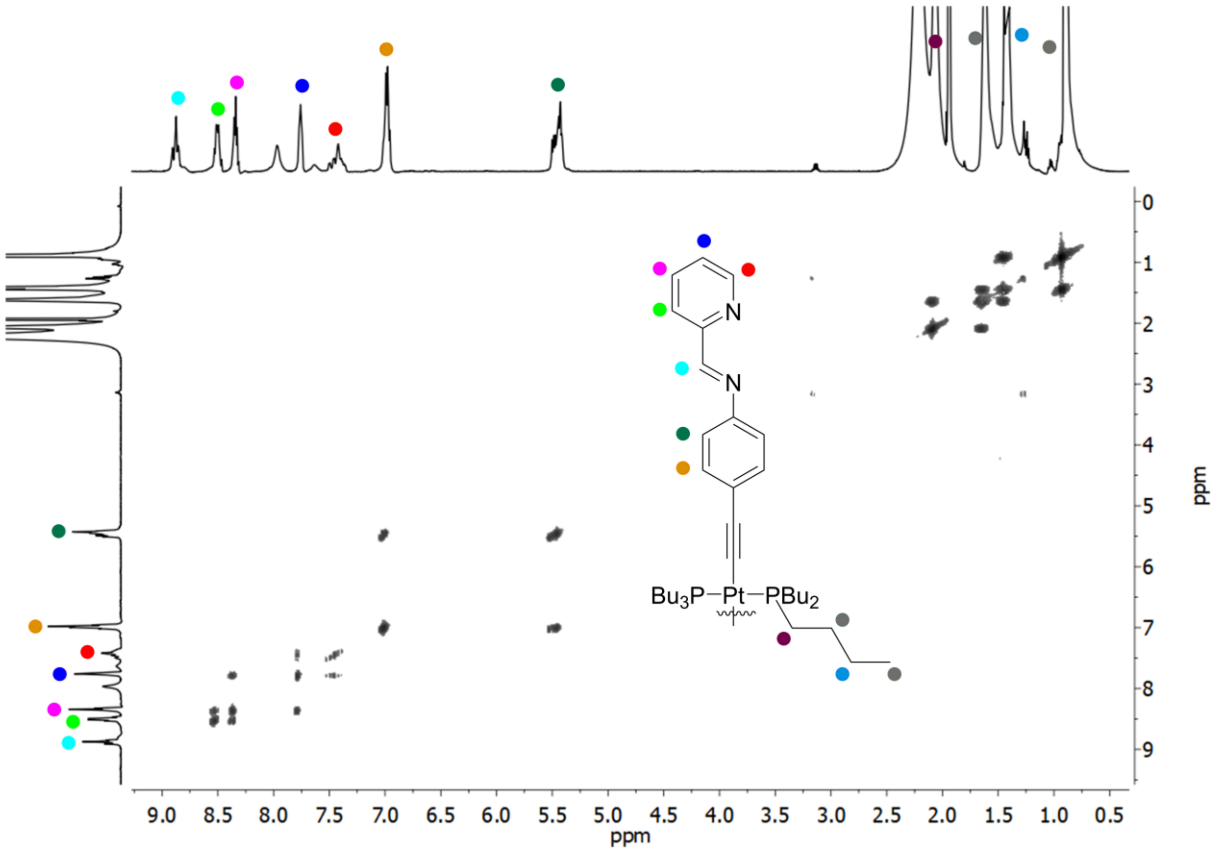


**Figure S5.** $^{1}H$-$^{1}H$-COSY spectrum of **4** (500 MHz, acetonitrile-*d*3, 298 K).

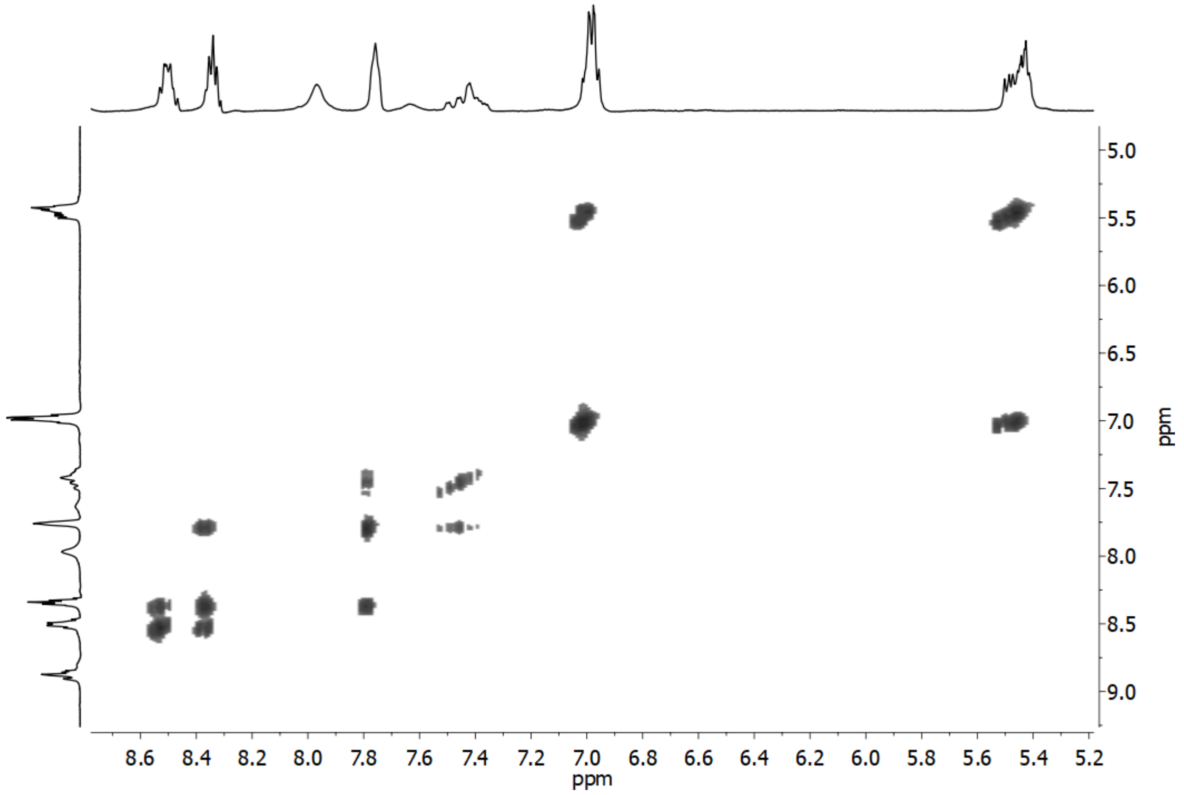


**Figure S6.** Aromatic region of the $^{1}H$-$^{1}H$-COSY spectrum of **4** (500 MHz, acetonitrile-*d*3, 298 K).

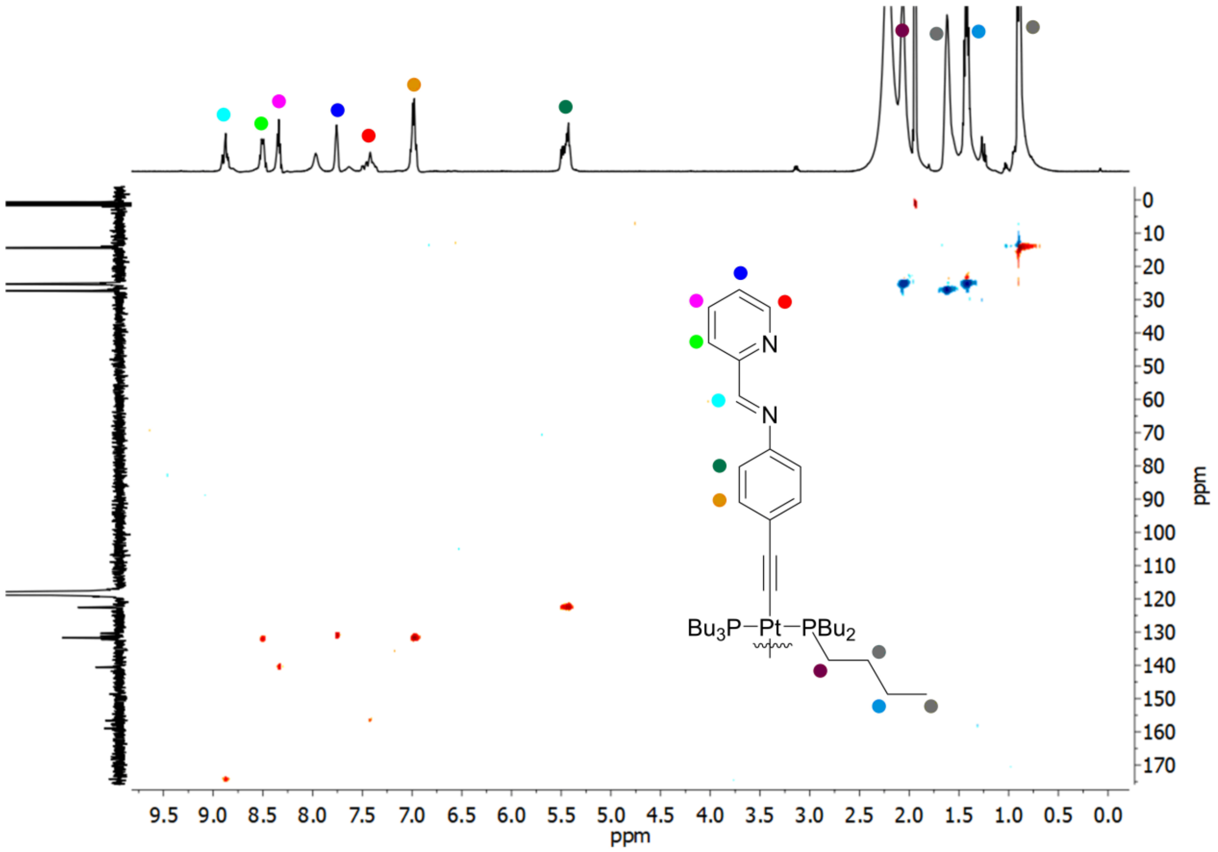


**Figure S7.** $^{1}$H-$^{13}$C-HSQC spectrum of **4** (500 MHz, 126 MHz, acetonitrile-$d_3$, 298 K). Blue signals = $CH_2$; red signals = CH, $CH_3$

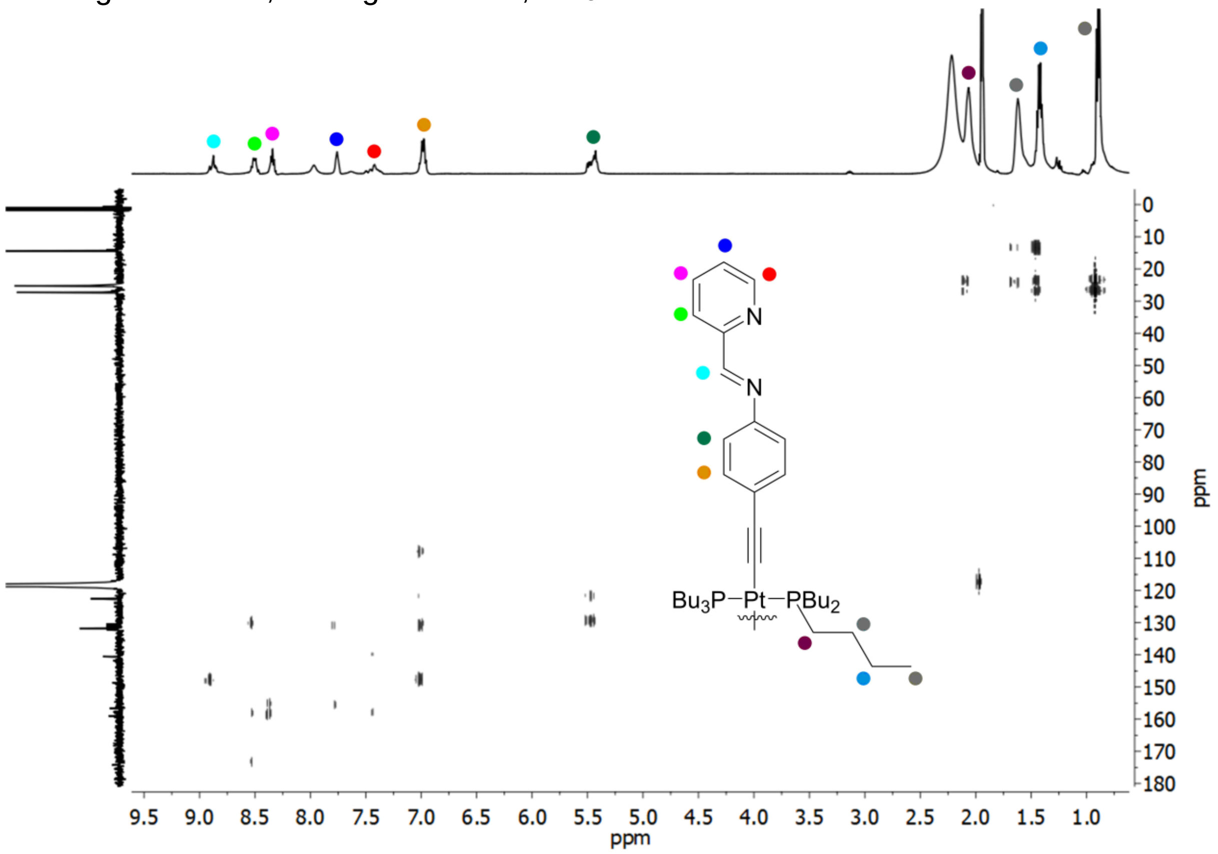


**Figure S8.** $^{1}$H-$^{13}$C-HMBC spectrum of **4** (500 MHz, 126 MHz, acetonitrile-$d_3$, 298 K).

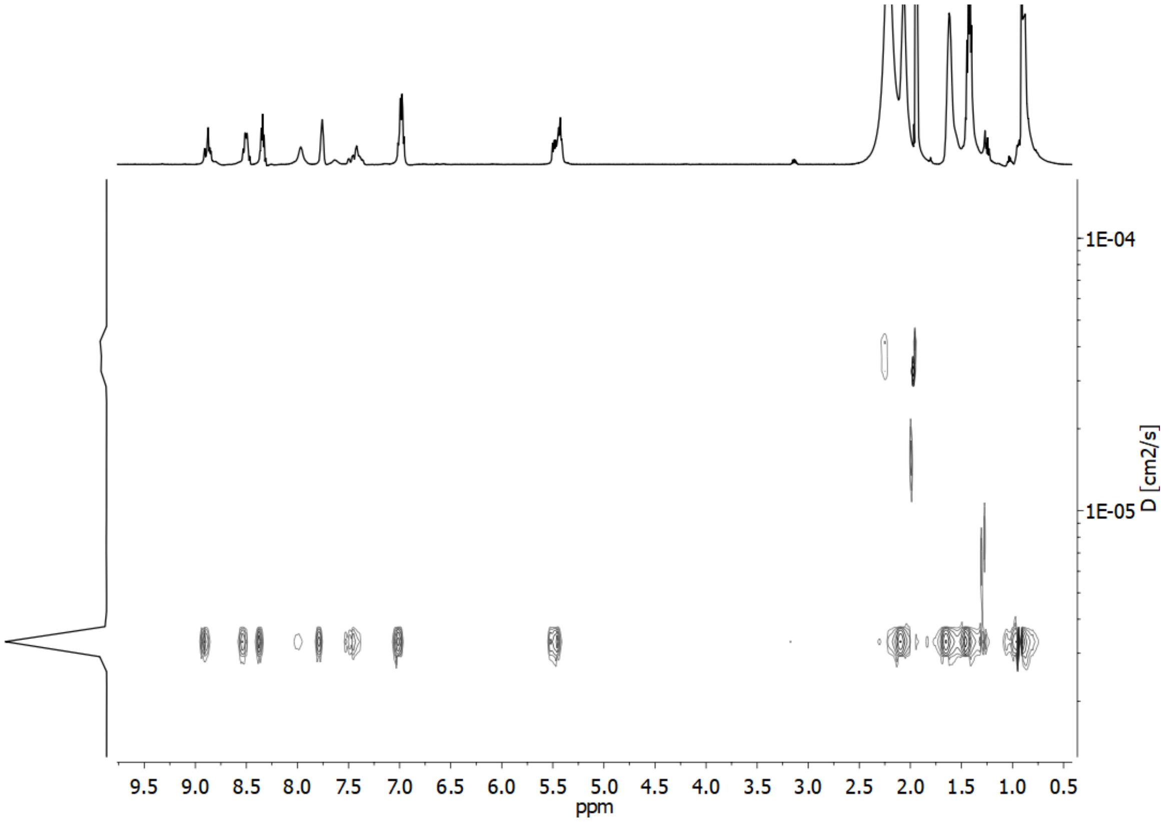


**Figure S9.** $^{1}$H-2D-DOSY spectrum of **4** (500 MHz, acetonitrile-$d_3$, 298 K, $\tau$ = 150 ms). $D$ = 3.33·10$^{-10}$ m$^2$/s, $d_h$ = 33 Å, $r_h$ = 1.65 nm.

## Selected isotopic pattern from the mass spectrum of metallosupramolecular tetrahedron 4

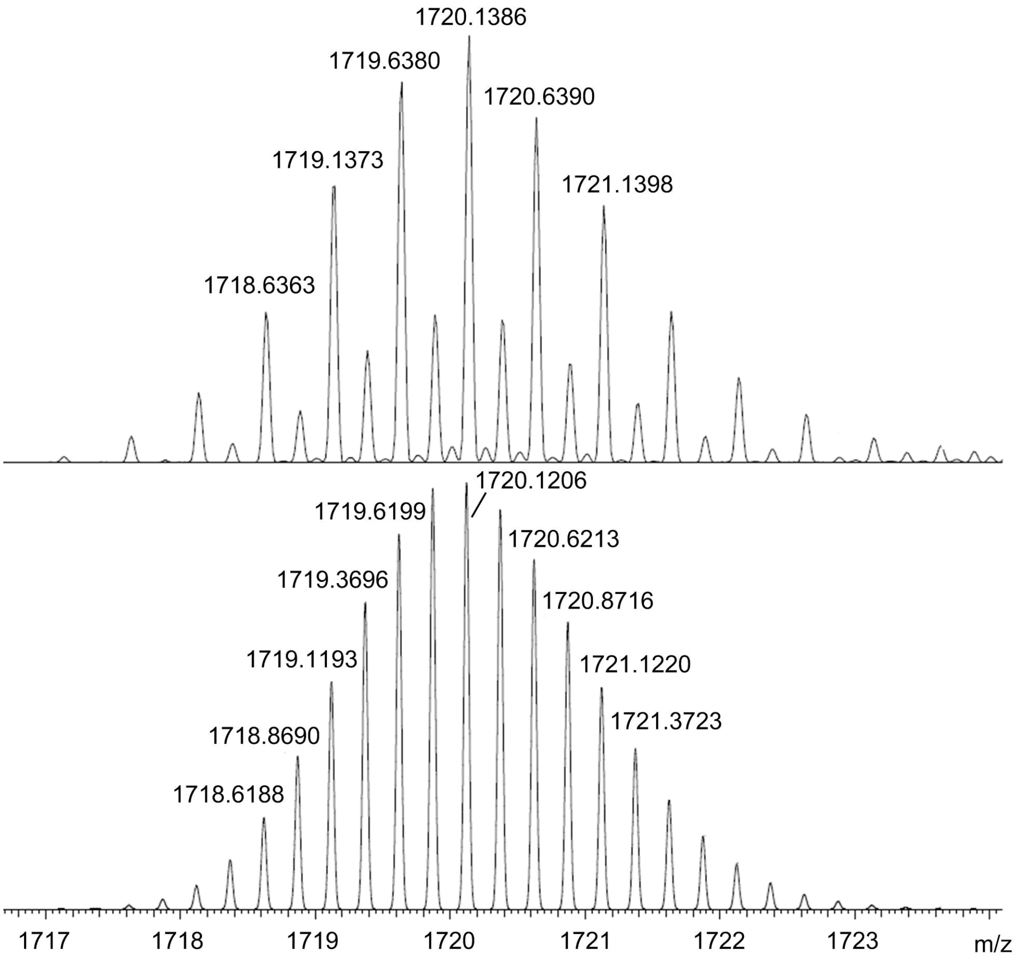


**Figure S10.** Isotopic pattern of the [**4** – 4 OTf]$^{4+}$ peak from the mass spectrum of **4**. Top: measured spectrum; bottom: calculated spectrum.

The isotopic pattern of the [**4** – 4 OTf]$^{4+}$ peak from the mass spectrum of **4** shows the presence of the expected four times positively charged species next to the doubly charged fragment [$Fe_2Pt_3L_6$ + 2 OTf]$^{2+}$. The superposition of these two peaks is a commonly found phenomenon with ESI mass spectrometry with rather labile metallosupramolecular architectures.

## Atom coordinates of energy-minimized cages 4

We performed computational calculations for all possible diastereomers of the cationic unit of metallosupramolecular cage **4**. Please note, that these calculations do not include any solvent molecules or counter anions. The models were minimized regarding symmetry considerations, and therefore, the calculations yield reasonable molecular structures of the presented cages and can be used to verify spectroscopic and spectrometric findings, e.g. the size and shape. However, these calculations do not give reasonable insights into energy differences between the diastereomers, and therefore, these are not discussed herein.

### (*all*-Δ)-*T*-4

| | x-coord. | y.coord. | z-coord. |
|---|---|---|---|
| Fe | 69.062 | 65.995 | -77.615 |
| N | 65.928 | 69.171 | -96.511 |
| C | 59.413 | 70.205 | -123.605 |
| C | 65.542 | 80.825 | -103.001 |
| C | 63.094 | 57.867 | -103.380 |
| C | 59.814 | 58.096 | -116.927 |
| C | 62.364 | 81.737 | -116.457 |
| H | 67.878 | 89.750 | -97.305 |
| H | 57.661 | 48.784 | -122.021 |
| H | 62.237 | 91.453 | -121.235 |
| H | 56.905 | 70.667 | -134.132 |
| N | 87.958 | 61.909 | -79.629 |
| C | 114.621 | 53.852 | -79.005 |
| C | 93.693 | 57.956 | -68.049 |
| C | 95.343 | 61.928 | -90.741 |
| C | 108.636 | 58.014 | -90.822 |
| C | 107.003 | 53.853 | -67.457 |
| H | 90.528 | 65.188 | -99.892 |
| H | 114.189 | 58.257 | -100.114 |
| H | 111.185 | 50.768 | -57.954 |
| H | 124.992 | 50.732 | -78.820 |
| N | 72.611 | 84.656 | -73.260 |
| C | 74.215 | 110.817 | -63.718 |
| C | 61.615 | 91.034 | -68.687 |
| C | 84.230 | 91.225 | -73.246 |
| C | 85.412 | 104.237 | -68.633 |
| C | 62.136 | 104.081 | -63.787 |
| H | 92.912 | 85.983 | -77.075 |
| H | 95.076 | 109.111 | -68.919 |
| H | 53.052 | 108.757 | -60.190 |
| H | 74.905 | 120.969 | -60.006 |
| C | 49.521 | 83.454 | -69.920 |
| H | 40.111 | 87.949 | -66.837 |
| C | 64.284 | 45.891 | -95.567 |
| H | 63.216 | 36.152 | -100.286 |
| C | 84.998 | 58.716 | -56.653 |
| H | 88.792 | 56.173 | -46.781 |
| N | 72.766 | 62.527 | -58.543 |
| N | 66.851 | 47.142 | -82.929 |
| N | 50.132 | 71.525 | -75.003 |
| C | 37.843 | 65.189 | -77.666 |
| C | 12.507 | 53.074 | -79.526 |
| C | 26.515 | 72.763 | -81.080 |
| C | 36.476 | 51.307 | -76.479 |
| C | 24.045 | 45.396 | -77.244 |
| C | 14.112 | 66.814 | -81.967 |
| H | 27.503 | 83.346 | -83.199 |
| H | 45.132 | 45.134 | -74.557 |
| H | 23.116 | 34.681 | -75.856 |
| H | 0.5440 | 72.825 | -84.442 |
| C | 70.181 | 35.401 | -75.838 |
| C | 76.688 | 11.714 | -62.102 |
| C | 78.483 | 25.772 | -81.753 |
| C | 65.533 | 33.219 | -62.843 |
| C | 68.623 | 21.570 | -56.166 |
| C | 81.687 | 14.165 | -74.994 |
| H | 82.672 | 27.630 | -91.579 |

| | | | |
|---|---|---|---|
| H | 59.203 | 40.531 | -58.041 |
| H | 64.736 | 19.928 | -46.175 |
| H | 88.156 | 0.6842 | -79.679 |
| C | 64.840 | 64.582 | -47.059 |
| C | 49.493 | 67.242 | -23.622 |
| C | 51.363 | 60.860 | -46.891 |
| C | 70.394 | 70.270 | -35.506 |
| C | 62.833 | 71.609 | -24.031 |
| C | 43.885 | 62.017 | -35.385 |
| H | 46.732 | 56.734 | -55.723 |
| H | 80.602 | 73.913 | -35.676 |
| H | 67.241 | 76.101 | -15.209 |
| H | 33.528 | 58.814 | -35.370 |
| C | 41.492 | 68.106 | -12.007 |
| C | 33.877 | 68.594 | -0.2502 |
| C | 79.360 | -0.0185 | -54.941 |
| C | 80.983 | -10.241 | -48.245 |
| C | -0.0263 | 47.035 | -79.106 |
| C | -11.178 | 41.713 | -78.160 |
| Fe | -105.865 | -33.387 | -55.087 |
| N | -122.777 | -36.666 | -46.080 |
| C | -144.669 | -40.991 | -29.372 |
| C | -135.096 | -33.328 | -49.973 |
| C | -121.156 | -42.261 | -33.883 |
| C | -131.919 | -44.512 | -25.314 |
| C | -146.222 | -35.351 | -41.963 |
| H | -136.164 | -28.920 | -59.821 |
| H | -130.139 | -49.025 | -15.630 |
| H | -155.999 | -32.501 | -45.639 |
| H | -153.211 | -42.646 | -22.920 |
| N | -105.483 | -50.645 | -63.999 |
| C | -100.536 | -75.061 | -76.491 |
| C | -93.922 | -52.767 | -70.668 |
| C | -114.504 | -60.467 | -63.583 |
| C | -112.418 | -72.726 | -69.695 |
| C | -91.172 | -64.891 | -76.980 |
| H | -123.729 | -58.509 | -58.233 |
| H | -120.098 | -80.334 | -69.098 |
| H | -81.773 | -66.136 | -82.215 |
| H | -98.672 | -84.569 | -81.335 |
| N | -113.944 | -24.124 | -70.165 |
| C | -123.394 | -0.7344 | -90.327 |
| C | -114.934 | -10.792 | -68.159 |
| C | -117.653 | -29.078 | -81.989 |
| C | -122.423 | -21.064 | -92.236 |
| C | -119.598 | -0.2168 | -78.074 |
| H | -116.837 | -39.803 | -83.355 |

| | | | |
|---|---|---|---|
| H | -125.343 | -25.618 | -101.615 |
| H | -120.227 | 0.8445 | -76.005 |
| H | -127.078 | -0.0876 | -98.195 |
| C | -111.266 | -0.6610 | -54.943 |
| H | -112.301 | 0.3834 | -52.099 |
| C | -107.632 | -46.075 | -30.977 |
| H | -105.381 | -51.358 | -21.740 |
| C | -85.244 | -41.351 | -71.034 |
| H | -75.981 | -41.788 | -76.715 |
| N | -88.922 | -30.652 | -64.701 |
| N | -98.466 | -43.308 | -39.711 |
| N | -106.983 | -15.603 | -46.641 |
| C | -105.237 | -11.619 | -33.244 |
| C | -100.633 | -0.1880 | -0.7258 |
| C | -114.042 | -0.2375 | -27.412 |
| C | -94.662 | -16.593 | -25.557 |
| C | -92.350 | -11.752 | -12.868 |
| C | -111.774 | 0.2390 | -14.665 |
| H | -122.805 | 0.0898 | -32.888 |
| H | -87.954 | -23.994 | -29.661 |
| H | -83.903 | -15.459 | -0.7171 |
| H | -118.590 | 0.9619 | -10.342 |
| C | -85.726 | -48.999 | -37.546 |
| C | -60.585 | -60.107 | -31.394 |
| C | -84.724 | -62.029 | -32.444 |
| C | -73.970 | -41.926 | -40.247 |
| C | -61.662 | -47.324 | -37.106 |
| C | -72.400 | -67.456 | -29.448 |
| H | -93.691 | -67.964 | -31.075 |
| H | -74.436 | -31.998 | -44.497 |
| H | -52.670 | -41.551 | -38.953 |
| H | -71.802 | -77.517 | -25.455 |
| C | -81.232 | -19.035 | -66.880 |
| C | -63.922 | 0.2761 | -71.079 |
| C | -78.540 | -0.9995 | -56.558 |
| C | -75.978 | -16.444 | -79.632 |
| C | -67.505 | -0.5758 | -81.665 |
| C | -70.021 | 0.0659 | -58.610 |
| H | -82.803 | -11.496 | -46.747 |
| H | -78.737 | -22.787 | -87.978 |
| H | -63.423 | -0.3967 | -91.545 |
| H | -67.858 | 0.7432 | -50.430 |
| C | -54.013 | 12.712 | -72.717 |
| C | -44.818 | 20.642 | -73.810 |
| C | -47.998 | -65.332 | -27.556 |
| C | -37.167 | -69.563 | -23.900 |
| C | -97.146 | 0.3750 | 0.5223 |

| | | | | | | | |
|---|---|---|---|---|---|---|---|
| C | -92.723 | 0.8790 | 15.405 | H | 43.903 | -91.675 | 68.299 |
| Fe | 67.219 | -101.212 | 32.283 | H | 55.437 | -70.370 | 32.848 |
| N | 76.347 | -101.639 | 49.451 | H | 49.460 | -49.267 | 43.531 |
| C | 89.161 | -97.964 | 73.927 | H | 37.881 | -70.448 | 79.019 |
| C | 76.207 | -111.228 | 58.728 | C | 78.028 | -75.458 | 19.370 |
| C | 82.895 | -90.128 | 52.153 | C | 81.367 | -57.514 | -0.2061 |
| C | 89.369 | -88.014 | 64.318 | C | 90.643 | -70.043 | 16.489 |
| C | 82.481 | -109.779 | 70.998 | C | 67.283 | -72.313 | 11.000 |
| H | 70.949 | -120.396 | 56.310 | C | 68.908 | -63.432 | 0.0593 |
| H | 94.500 | -78.631 | 66.035 | C | 92.269 | -61.264 | 0.5962 |
| H | 82.095 | -117.898 | 78.150 | H | 99.226 | -73.004 | 22.413 |
| H | 94.124 | -96.593 | 83.456 | H | 57.510 | -76.547 | 12.815 |
| N | 82.535 | -108.202 | 22.531 | H | 60.381 | -60.902 | -0.5600 |
| C | 103.177 | -115.417 | 0.5230 | H | 102.081 | -57.169 | 0.3858 |
| C | 80.770 | -107.832 | 0.9138 | C | 45.530 | -99.262 | 10.006 |
| C | 94.349 | -112.193 | 27.286 | C | 19.797 | -93.500 | 0.0018 |
| C | 104.827 | -115.892 | 19.009 | C | 37.199 | -90.139 | 16.562 |
| C | 90.934 | -111.337 | 0.0256 | C | 40.382 | -106.237 | -0.1049 |
| H | 95.522 | -112.497 | 38.059 | C | 27.811 | -103.366 | -0.5963 |
| H | 114.184 | -119.109 | 23.406 | C | 24.642 | -87.323 | 11.657 |
| H | 89.058 | -110.870 | -10.401 | H | 40.680 | -84.905 | 25.337 |
| H | 111.235 | -118.228 | -0.1442 | H | 46.234 | -114.089 | -0.5694 |
| N | 58.747 | -118.536 | 34.524 | H | 24.087 | -108.732 | -14.610 |
| C | 43.012 | -141.277 | 38.089 | H | 18.440 | -80.047 | 16.744 |
| C | 46.829 | -117.769 | 40.860 | C | 0.7400 | -89.369 | -0.5398 |
| C | 62.822 | -130.430 | 30.047 | C | -0.2933 | -84.718 | -0.9890 |
| C | 55.297 | -141.952 | 31.654 | C | 82.542 | -48.024 | -12.488 |
| C | 38.753 | -128.974 | 42.764 | C | 82.877 | -39.734 | -21.416 |
| H | 72.429 | -130.820 | 25.038 | C | 38.504 | -46.026 | 67.545 |
| H | 59.110 | -151.353 | 27.869 | C | 33.945 | -35.438 | 71.531 |
| H | 29.290 | -127.877 | 47.916 | Fe | -28.054 | 65.767 | 102.473 |
| H | 36.948 | -150.147 | 39.458 | N | -19.232 | 82.725 | 106.120 |
| C | 43.649 | -104.657 | 45.727 | C | -0.3514 | 105.633 | 108.651 |
| H | 34.614 | -103.107 | 51.579 | C | -24.692 | 94.423 | 109.512 |
| C | 82.904 | -80.754 | 41.290 | C | -0.5893 | 82.227 | 104.016 |
| H | 88.469 | -71.451 | 42.152 | C | 0.2198 | 93.528 | 105.166 |
| C | 67.545 | -104.085 | 0.5069 | C | -17.210 | 106.003 | 110.902 |
| H | 65.063 | -103.945 | -0.5517 | H | -35.393 | 94.603 | 111.234 |
| N | 58.827 | -101.202 | 14.231 | H | 12.842 | 92.629 | 103.376 |
| N | 76.336 | -83.861 | 30.561 | H | -22.158 | 115.207 | 113.736 |
| N | 51.859 | -94.980 | 43.076 | H | 0.2541 | 114.558 | 109.654 |
| C | 49.469 | -82.653 | 49.515 | N | -26.389 | 59.161 | 120.664 |
| C | 42.828 | -58.270 | 61.948 | C | -24.332 | 45.919 | 145.115 |
| C | 44.781 | -82.412 | 62.738 | C | -31.241 | 46.631 | 122.166 |
| C | 51.519 | -70.498 | 42.912 | C | -20.623 | 65.056 | 131.156 |
| C | 48.140 | -58.566 | 48.945 | C | -19.439 | 58.807 | 143.468 |
| C | 41.555 | -70.455 | 68.824 | C | -30.333 | 39.774 | 134.271 |

| | | | |
|---|---|---|---|
| H | -16.842 | 75.118 | 129.740 |
| H | -14.712 | 64.078 | 151.660 |
| H | -34.386 | 29.757 | 135.007 |
| H | -23.519 | 40.827 | 154.642 |
| N | -45.846 | 73.073 | 105.217 |
| C | -71.927 | 82.914 | 105.039 |
| C | -51.370 | 77.773 | 93.818 |
| C | -53.128 | 73.306 | 116.397 |
| C | -66.108 | 78.149 | 116.708 |
| C | -64.401 | 82.717 | 93.433 |
| H | -48.474 | 69.544 | 125.437 |
| H | -71.547 | 78.155 | 126.070 |
| H | -68.409 | 86.380 | 84.061 |
| H | -82.064 | 86.733 | 105.028 |
| C | -42.567 | 77.534 | 82.494 |
| H | -46.050 | 81.381 | 72.936 |
| C | -0.0913 | 69.126 | 101.020 |
| H | 0.9764 | 67.690 | 99.538 |
| C | -37.691 | 41.429 | 110.456 |
| H | -42.532 | 31.697 | 110.818 |
| N | -37.674 | 48.694 | 99.725 |
| N | -0.9347 | 59.298 | 100.326 |
| N | -30.615 | 72.777 | 84.060 |
| C | -21.842 | 73.854 | 73.065 |
| C | -0.6142 | 74.546 | 49.709 |
| C | -22.496 | 84.824 | 64.340 |
| C | -12.574 | 63.734 | 70.373 |
| C | -0.4999 | 63.987 | 58.880 |
| C | -14.781 | 85.142 | 52.892 |
| H | -28.930 | 93.227 | 66.680 |
| H | -11.500 | 55.388 | 77.111 |
| H | 0.1859 | 55.845 | 56.806 |
| H | -15.442 | 93.663 | 46.223 |
| C | -0.3923 | 46.379 | 98.789 |
| C | 0.8305 | 21.720 | 92.861 |
| C | 0.8552 | 43.069 | 104.315 |
| C | -10.642 | 36.704 | 91.239 |
| C | -0.4579 | 24.731 | 88.173 |
| C | 14.508 | 30.958 | 101.429 |
| H | 13.495 | 49.963 | 111.061 |
| H | -20.530 | 38.679 | 87.399 |
| H | -0.9765 | 17.576 | 81.898 |
| H | 24.157 | 28.569 | 105.749 |
| C | -45.648 | 44.216 | 88.970 |
| C | -61.153 | 34.055 | 67.769 |
| C | -41.350 | 45.507 | 75.729 |
| C | -58.110 | 38.272 | 91.447 |
| C | -65.697 | 33.304 | 81.042 |
| C | -48.873 | 40.420 | 65.367 |
| H | -31.913 | 50.239 | 73.487 |
| H | -61.945 | 37.851 | 101.577 |
| H | -75.316 | 28.760 | 83.113 |
| H | -45.227 | 41.288 | 55.189 |
| C | -68.538 | 28.715 | 56.959 |
| C | -74.554 | 24.210 | 47.375 |
| C | 14.489 | 0.9678 | 88.797 |
| C | 19.002 | -0.0801 | 84.522 |
| C | 0.1477 | 74.078 | 37.811 |
| C | 0.8363 | 72.643 | 27.877 |
| Pt | -83.806 | 16.682 | 31.369 |
| Pt | -28.302 | 31.706 | -76.032 |
| Pt | -19.871 | -76.913 | -17.035 |
| Pt | 21.007 | 70.434 | 12.561 |
| Pt | 82.135 | -25.505 | -35.385 |
| Pt | 26.267 | -18.172 | 77.886 |
| P | 59.224 | -24.459 | -34.064 |
| P | 104.974 | -25.262 | -38.005 |
| P | 0.9668 | 87.193 | 0.1683 |
| P | 30.018 | 52.736 | 24.052 |
| P | -63.559 | 17.517 | 20.484 |
| P | -104.200 | 16.260 | 41.965 |
| P | -12.184 | -55.923 | -11.672 |
| P | -28.369 | -97.762 | -21.769 |
| P | 0.4906 | -25.435 | 73.211 |
| P | 46.798 | -10.006 | 84.045 |
| P | -18.010 | 12.455 | -82.955 |
| P | -38.873 | 50.405 | -67.979 |
| C | -44.815 | -100.088 | -13.628 |
| H | -42.935 | -102.959 | -0.3234 |
| H | -49.351 | -90.134 | -13.282 |
| C | -54.876 | -109.797 | -19.933 |
| H | -50.788 | -119.926 | -20.169 |
| H | -56.964 | -106.765 | -30.234 |
| C | -67.950 | -109.807 | -11.933 |
| H | -65.868 | -112.696 | -0.1582 |
| H | -72.098 | -99.660 | -11.788 |
| C | -78.125 | -119.442 | -17.999 |
| H | -74.197 | -129.610 | -18.032 |
| H | -80.458 | -116.584 | -28.265 |
| H | -87.328 | -119.298 | -12.166 |
| C | -31.210 | -98.913 | -40.008 |
| H | -22.724 | -93.825 | -44.684 |
| H | -39.874 | -92.477 | -41.864 |
| C | -33.304 | -112.506 | -46.896 |

| | | | | | | | |
|---|---|---|---|---|---|---|---|
| H | -39.622 | -110.853 | -55.686 | H | -19.463 | -0.1162 | -0.9860 |
| H | -38.666 | -119.412 | -40.354 | C | -14.278 | -53.011 | 0.6481 |
| C | -20.126 | -118.848 | -51.443 | H | -0.7863 | -60.139 | 11.730 |
| H | -15.319 | -112.284 | -58.774 | H | -11.167 | -42.979 | 0.9451 |
| H | -13.308 | -119.815 | -42.937 | C | -28.870 | -55.658 | 10.505 |
| C | -22.492 | -132.609 | -57.628 | H | -31.138 | -66.166 | 0.8527 |
| H | -13.031 | -136.885 | -60.932 | H | -35.623 | -49.709 | 0.4281 |
| H | -29.155 | -131.846 | -66.223 | C | -31.605 | -52.651 | 25.270 |
| H | -26.992 | -139.346 | -50.327 | H | -24.651 | -58.362 | 31.498 |
| H | -0.8965 | -111.981 | -22.678 | H | -41.723 | -56.090 | 27.626 |
| C | -17.729 | -111.753 | -16.124 | C | -30.510 | -37.789 | 28.692 |
| H | -13.995 | -108.699 | -0.6287 | H | -20.308 | -34.156 | 27.368 |
| C | -23.748 | -125.804 | -14.920 | H | -33.364 | -36.158 | 39.090 |
| H | -28.025 | -129.005 | -24.443 | H | -37.132 | -31.893 | 22.326 |
| H | -31.775 | -125.733 | -0.7493 | C | 50.596 | 0.4641 | 73.319 |
| C | -12.971 | -135.837 | -10.659 | H | 40.841 | 0.8704 | 70.558 |
| H | -0.8750 | -132.753 | -0.1031 | H | 54.747 | 0.0435 | 64.104 |
| H | -0.4865 | -135.765 | -18.023 | C | 59.639 | 16.105 | 78.107 |
| C | -18.735 | -149.931 | -0.9507 | H | 63.960 | 20.875 | 69.241 |
| H | -10.937 | -156.909 | -0.6470 | H | 67.954 | 12.203 | 84.015 |
| H | -22.771 | -153.190 | -19.096 | C | 52.243 | 26.757 | 86.275 |
| H | -26.729 | -150.201 | -0.2098 | H | 59.691 | 33.552 | 90.525 |
| C | 0.5597 | -52.237 | -15.901 | H | 46.913 | 22.092 | 94.608 |
| H | 0.6883 | -41.753 | -18.713 | C | 42.343 | 34.895 | 77.935 |
| H | 0.7406 | -58.077 | -24.977 | H | 38.069 | 42.881 | 84.010 |
| C | 16.236 | -56.135 | -0.5512 | H | 34.150 | 28.670 | 74.306 |
| H | 25.152 | -59.585 | -10.860 | H | 47.347 | 39.389 | 69.340 |
| H | 12.681 | -64.502 | 0.0543 | C | 61.041 | -21.614 | 82.122 |
| C | 20.207 | -44.495 | 0.3593 | H | 58.940 | -27.450 | 73.096 |
| H | 11.770 | -41.650 | 0.9959 | H | 60.576 | -28.684 | 90.470 |
| H | 22.785 | -35.807 | -0.2560 | C | 75.187 | -15.793 | 81.058 |
| C | 32.178 | -48.115 | 12.367 | H | 77.542 | -0.9807 | 89.889 |
| H | 40.904 | -50.322 | 0.6201 | H | 75.842 | -0.9250 | 72.312 |
| H | 29.971 | -56.892 | 18.451 | C | 85.469 | -27.086 | 79.739 |
| H | 34.634 | -39.823 | 19.009 | H | 83.171 | -33.053 | 70.838 |
| C | -21.186 | -41.838 | -19.777 | H | 84.718 | -33.682 | 88.446 |
| H | -16.505 | -40.112 | -29.521 | C | 99.666 | -21.554 | 78.722 |
| H | -31.203 | -45.710 | -21.890 | H | 106.800 | -29.741 | 77.804 |
| C | -22.842 | -28.471 | -12.448 | H | 102.149 | -15.772 | 87.624 |
| H | -27.664 | -30.068 | -0.2763 | H | 100.642 | -15.087 | 69.993 |
| H | -29.690 | -22.368 | -18.440 | C | 46.291 | -0.5213 | 101.843 |
| C | -0.9882 | -20.608 | -10.406 | H | 41.176 | -13.560 | 106.746 |
| H | -0.2992 | -26.300 | -0.4098 | H | 39.459 | 0.3299 | 102.620 |
| H | -0.4994 | -18.980 | -20.068 | C | 59.340 | -0.2261 | 109.349 |
| C | -12.707 | -0.7152 | -0.3739 | H | 65.094 | 0.5362 | 104.045 |
| H | -0.3445 | -0.1574 | -0.2347 | H | 65.423 | -11.337 | 109.817 |
| H | -17.317 | -0.8668 | 0.6031 | C | 56.603 | 0.2771 | 123.581 |

| | | | |
|---|---|---|---|
| H | 66.111 | 0.6095 | 127.854 |
| H | 49.952 | 11.464 | 123.126 |
| C | 50.530 | -0.7911 | 132.651 |
| H | 49.613 | -0.4023 | 142.789 |
| H | 56.884 | -16.769 | 132.909 |
| H | 40.605 | -10.840 | 129.200 |
| C | 0.0847 | -38.904 | 85.142 |
| H | 0.1017 | -34.394 | 95.111 |
| H | 0.9509 | -45.580 | 84.820 |
| C | -12.056 | -47.035 | 83.388 |
| H | -13.770 | -49.346 | 72.832 |
| H | -20.547 | -41.105 | 86.899 |
| C | -11.537 | -60.141 | 91.348 |
| H | -0.8292 | -58.075 | 101.591 |
| H | -21.667 | -64.245 | 91.836 |
| C | -0.2295 | -70.526 | 85.004 |
| H | 0.8054 | -67.068 | 84.851 |
| H | -0.5379 | -72.636 | 74.748 |
| H | -0.2700 | -79.796 | 90.719 |
| C | -0.8004 | -12.315 | 75.351 |
| H | -0.4237 | -0.3546 | 69.977 |
| H | -0.7828 | -0.9626 | 85.961 |
| C | -22.461 | -14.941 | 70.998 |
| H | -22.875 | -16.658 | 60.196 |
| H | -26.386 | -23.856 | 75.951 |
| C | -31.288 | -0.2910 | 74.554 |
| H | -27.419 | 0.6022 | 69.518 |
| H | -30.766 | -0.1110 | 85.342 |
| C | -45.842 | -0.5147 | 70.489 |
| H | -46.599 | -0.7034 | 59.768 |
| H | -51.782 | 0.3684 | 72.865 |
| H | -50.030 | -13.698 | 75.799 |
| H | 0.2792 | -24.786 | 48.976 |
| C | 0.1001 | -32.523 | 56.498 |
| H | -0.9623 | -35.074 | 55.944 |
| C | 0.9442 | -44.926 | 53.281 |
| H | 19.325 | -41.783 | 49.840 |
| H | 10.902 | -50.878 | 62.336 |
| C | 0.2828 | -53.762 | 42.697 |
| H | 0.2490 | -48.466 | 33.116 |
| H | -0.7475 | -55.919 | 45.707 |
| C | 10.388 | -66.929 | 41.098 |
| H | 20.717 | -65.096 | 38.099 |
| H | 0.5557 | -73.112 | 33.529 |
| H | 10.488 | -72.436 | 50.516 |
| C | -103.319 | 20.976 | 59.817 |
| H | -94.909 | 15.112 | 63.685 |
| H | -100.035 | 31.413 | 60.186 |
| C | -112.401 | -0.0300 | 41.262 |
| H | -108.533 | -0.6281 | 49.573 |
| H | -108.833 | -0.5085 | 32.089 |
| C | -115.255 | 18.985 | 69.266 |
| H | -111.394 | 19.916 | 79.479 |
| H | -119.215 | 0.8845 | 68.248 |
| C | -126.483 | 29.210 | 67.343 |
| H | -122.267 | 39.319 | 67.499 |
| H | -131.246 | 27.736 | 57.606 |
| C | -137.042 | 27.872 | 78.298 |
| H | -132.619 | 29.596 | 88.113 |
| H | -141.426 | 17.891 | 78.161 |
| H | -144.973 | 35.175 | 76.726 |
| C | -127.749 | -0.0602 | 41.345 |
| H | -131.641 | 0.4699 | 50.071 |
| H | -131.554 | 0.4443 | 32.414 |
| C | -132.801 | -15.068 | 41.560 |
| H | -128.898 | -20.131 | 50.448 |
| H | -128.987 | -20.364 | 32.761 |
| C | -148.064 | -15.558 | 41.674 |
| H | -151.977 | -10.476 | 50.489 |
| H | -151.452 | -25.913 | 41.849 |
| H | -152.119 | -10.714 | 32.784 |
| H | -125.767 | 27.679 | 37.673 |
| C | -115.719 | 27.875 | 33.410 |
| H | -116.499 | 24.378 | 23.066 |
| C | -110.310 | 42.232 | 33.441 |
| H | -100.225 | 42.320 | 29.183 |
| H | -109.557 | 45.865 | 43.737 |
| C | -119.445 | 51.508 | 25.379 |
| H | -120.132 | 47.846 | 15.082 |
| H | -129.507 | 51.292 | 29.675 |
| C | -114.184 | 65.842 | 25.392 |
| H | -120.833 | 72.252 | 19.610 |
| H | -104.216 | 66.272 | 20.973 |
| H | -113.635 | 69.700 | 35.579 |
| C | -52.852 | 0.3838 | 26.942 |
| H | -56.956 | -0.5268 | 22.465 |
| H | -55.200 | 0.3170 | 37.607 |
| C | -37.590 | 0.3872 | 25.197 |
| H | -34.747 | 0.8188 | 15.572 |
| H | -34.175 | -0.6542 | 25.186 |
| C | -30.431 | 11.299 | 36.502 |
| H | -32.892 | 0.6538 | 46.038 |
| H | -33.900 | 21.653 | 37.032 |
| C | -15.289 | 11.179 | 34.570 |

| | | | |
|---|---|---|---|
| H | -11.522 | 0.0942 | 34.312 |
| H | -10.467 | 16.449 | 42.807 |
| H | -12.566 | 16.111 | 25.226 |
| C | -64.453 | 15.313 | 0.2100 |
| H | -66.987 | 25.054 | -0.2205 |
| H | -73.251 | 0.8986 | 0.0564 |
| C | -52.801 | 0.9145 | -0.5700 |
| H | -43.945 | 15.541 | -0.5151 |
| H | -50.196 | -0.0598 | -0.1456 |
| C | -56.843 | 0.7328 | -20.382 |
| H | -59.342 | 17.093 | -24.652 |
| H | -65.837 | 0.1096 | -20.928 |
| C | -45.695 | 0.0867 | -28.565 |
| H | -36.453 | 0.6597 | -27.683 |
| H | -48.471 | 0.0408 | -39.096 |
| H | -43.822 | -0.9291 | -25.069 |
| H | -62.746 | 41.217 | 22.314 |
| C | -54.883 | 33.651 | 23.129 |
| H | -51.721 | 33.792 | 33.600 |
| C | -43.211 | 37.552 | 13.965 |
| H | -47.084 | 40.150 | 0.4062 |
| H | -36.356 | 29.138 | 12.696 |
| C | -35.186 | 49.367 | 19.577 |
| H | -26.691 | 51.193 | 12.904 |
| H | -31.143 | 46.654 | 29.393 |
| C | -43.395 | 62.189 | 20.825 |
| H | -48.000 | 64.733 | 11.272 |
| H | -36.930 | 70.427 | 23.861 |
| H | -51.270 | 61.084 | 28.283 |
| C | 0.5366 | 101.138 | 12.967 |
| H | 14.107 | 102.553 | 19.408 |
| H | -0.2591 | 97.444 | 19.504 |
| C | 18.270 | 94.543 | -12.964 |
| H | 24.884 | 102.453 | -0.9291 |
| H | 24.832 | 86.597 | -16.654 |
| C | 0.1509 | 114.682 | 0.6873 |
| H | 10.075 | 118.700 | 0.1389 |
| H | -0.6722 | 113.524 | -0.0222 |
| C | -0.2564 | 124.742 | 17.712 |
| H | 0.5214 | 125.175 | 25.401 |
| H | -0.3215 | 134.631 | 13.083 |
| C | -16.007 | 121.408 | 24.162 |
| H | -18.795 | 129.297 | 31.143 |
| H | -15.536 | 111.992 | 29.669 |
| H | -23.801 | 120.586 | 16.572 |
| C | 10.027 | 99.791 | -24.773 |
| H | 0.3822 | 108.246 | -21.696 |
| H | 0.3396 | 91.910 | -28.476 |
| C | 19.408 | 104.188 | -36.081 |
| H | 25.686 | 112.435 | -32.564 |
| H | 26.036 | 95.861 | -38.725 |
| C | 11.612 | 108.593 | -48.450 |
| H | 18.495 | 111.926 | -56.222 |
| H | 0.4890 | 116.821 | -46.012 |
| H | 0.5699 | 100.311 | -52.384 |
| H | -0.4135 | 74.734 | -13.757 |
| C | -0.6311 | 80.085 | -0.4460 |
| H | -0.9085 | 72.430 | 0.2852 |
| C | -18.269 | 89.456 | -0.6491 |
| H | -15.656 | 97.639 | -13.242 |
| H | -21.145 | 93.813 | 0.3120 |
| C | -30.159 | 81.771 | -12.343 |
| H | -27.573 | 78.288 | -22.400 |
| H | -32.177 | 72.934 | -0.6189 |
| C | -42.663 | 90.505 | -13.006 |
| H | -40.813 | 99.371 | -19.074 |
| H | -45.616 | 93.713 | -0.3011 |
| H | -50.898 | 84.911 | -17.445 |
| C | 32.888 | 55.370 | 42.168 |
| H | 39.888 | 47.986 | 46.179 |
| H | 23.292 | 53.883 | 47.218 |
| C | 37.882 | 69.528 | 45.354 |
| H | 47.840 | 71.030 | 41.100 |
| H | 31.190 | 76.818 | 40.710 |
| C | 38.292 | 71.842 | 60.484 |
| H | 45.108 | 64.607 | 65.083 |
| H | 28.305 | 70.115 | 64.669 |
| C | 42.862 | 86.028 | 63.806 |
| H | 52.853 | 87.842 | 59.842 |
| H | 43.102 | 87.447 | 74.610 |
| H | 36.029 | 93.345 | 59.481 |
| C | 17.861 | 38.734 | 23.127 |
| H | 15.536 | 37.833 | 12.468 |
| H | 0.8674 | 42.290 | 27.891 |
| C | 21.375 | 24.755 | 28.451 |
| H | 31.390 | 21.818 | 25.170 |
| H | 14.312 | 17.710 | 23.900 |
| C | 20.360 | 23.453 | 43.683 |
| H | 28.449 | 29.040 | 48.470 |
| H | 10.879 | 27.750 | 47.101 |
| C | 21.167 | 0.8836 | 48.082 |
| H | 20.091 | 0.8120 | 58.906 |
| H | 30.766 | 0.4489 | 45.271 |
| H | 13.231 | 0.2943 | 43.460 |

| | | | |
|---|---|---|---|
| H | 43.753 | 42.544 | 0.7220 |
| C | 45.712 | 45.355 | 17.624 |
| H | 48.451 | 36.244 | 23.019 |
| C | 57.410 | 55.284 | 17.966 |
| H | 54.107 | 64.940 | 14.023 |
| H | 60.629 | 56.816 | 28.305 |
| C | 69.179 | 50.139 | 0.9625 |
| H | 65.927 | 48.802 | -0.0755 |
| H | 72.326 | 40.357 | 13.413 |
| C | 80.951 | 59.856 | 10.087 |
| H | 77.982 | 69.628 | 0.6255 |
| H | 89.163 | 56.082 | 0.3994 |
| H | 84.494 | 61.090 | 20.323 |
| C | 109.207 | -30.019 | -55.447 |
| H | 99.616 | -29.348 | -60.660 |
| H | 111.914 | -40.618 | -55.530 |
| C | 119.487 | -21.969 | -63.607 |
| H | 119.075 | -11.380 | -60.896 |
| H | 116.494 | -22.684 | -74.119 |
| C | 133.826 | -27.159 | -62.324 |
| H | 134.099 | -37.751 | -65.076 |
| H | 137.204 | -26.332 | -51.958 |
| C | 143.326 | -19.286 | -71.325 |
| H | 153.463 | -23.142 | -70.289 |
| H | 143.319 | -0.8730 | -68.575 |
| H | 140.325 | -20.169 | -81.769 |
| C | 111.058 | -0.8122 | -34.708 |
| H | 105.243 | -0.4651 | -26.104 |
| H | 107.813 | -0.1973 | -43.164 |
| C | 125.925 | -0.5756 | -31.864 |
| H | 128.805 | -11.050 | -22.740 |
| H | 132.044 | -0.9596 | -40.048 |
| C | 128.713 | 0.9215 | -30.125 |
| H | 122.690 | 13.093 | -21.838 |
| H | 125.680 | 14.534 | -39.213 |
| C | 143.520 | 11.763 | -27.380 |
| H | 146.645 | 0.6659 | -18.267 |
| H | 145.310 | 22.444 | -26.171 |
| H | 149.612 | 0.8128 | -35.658 |
| H | 107.809 | -45.628 | -25.986 |
| C | 114.008 | -36.618 | -26.566 |
| H | 113.596 | -32.049 | -16.620 |
| C | 128.402 | -40.781 | -29.774 |
| H | 128.717 | -45.747 | -39.510 |
| H | 134.900 | -32.015 | -30.256 |
| C | 133.680 | -50.362 | -19.037 |
| H | 127.183 | -59.170 | -18.534 |

| | | | |
|---|---|---|---|
| H | 133.324 | -45.406 | -0.9276 |
| C | 148.005 | -54.684 | -22.096 |
| H | 148.481 | -59.806 | -31.709 |
| H | 151.583 | -61.469 | -14.357 |
| H | 154.610 | -46.018 | -22.447 |
| C | 54.947 | -0.8204 | -26.181 |
| H | 56.907 | -0.0779 | -33.981 |
| H | 62.573 | -0.6464 | -18.542 |
| C | 41.049 | -0.5744 | -20.093 |
| H | 33.447 | -11.935 | -24.938 |
| H | 38.300 | 0.4708 | -21.881 |
| C | 40.901 | -0.8336 | -0.4993 |
| H | 48.374 | -0.1958 | -0.0159 |
| H | 43.646 | -18.737 | -0.2977 |
| C | 27.156 | -0.5451 | 0.1012 |
| H | 19.586 | -11.817 | -0.3590 |
| H | 24.363 | 0.4969 | -0.0627 |
| H | 27.235 | -0.7376 | 11.745 |
| C | 51.118 | -23.788 | -50.647 |
| H | 53.804 | -33.101 | -55.724 |
| H | 56.102 | -15.813 | -56.235 |
| C | 35.925 | -21.831 | -51.309 |
| H | 33.452 | -11.357 | -49.307 |
| H | 30.910 | -27.912 | -43.717 |
| C | 30.686 | -25.841 | -65.138 |
| H | 33.565 | -36.195 | -67.213 |
| H | 35.315 | -19.502 | -72.767 |
| C | 15.502 | -24.561 | -65.935 |
| H | 11.973 | -27.710 | -75.754 |
| H | 12.517 | -14.200 | -64.321 |
| H | 10.697 | -30.774 | -58.359 |
| C | 49.729 | -37.356 | -24.653 |
| H | 55.724 | -39.617 | -15.781 |
| H | 40.170 | -33.383 | -21.147 |
| C | 46.939 | -50.395 | -32.271 |
| H | 39.962 | -48.444 | -40.471 |
| H | 41.944 | -57.290 | -25.362 |
| C | 59.512 | -57.120 | -37.815 |
| H | 66.365 | -59.412 | -29.612 |
| H | 64.709 | -50.214 | -44.536 |
| C | 56.041 | -69.934 | -45.344 |
| H | 65.116 | -74.557 | -49.220 |
| H | 49.388 | -67.784 | -53.714 |
| H | 51.083 | -77.032 | -38.703 |
| C | -27.740 | 0.1525 | -94.204 |
| H | -36.413 | -0.2076 | -88.592 |
| H | -22.006 | -0.7246 | -97.328 |

| | | | |
|---|---|---|---|
| C | -32.612 | 0.9411 | -106.450 |
| H | -24.018 | 13.001 | -112.193 |
| H | -38.181 | 18.192 | -103.029 |
| C | -41.557 | 0.0803 | -115.406 |
| H | -49.963 | -0.3016 | -109.498 |
| H | -35.861 | -0.7817 | -119.025 |
| C | -46.869 | 0.8841 | -127.251 |
| H | -38.627 | 12.609 | -133.310 |
| H | -52.780 | 17.319 | -123.762 |
| H | -53.178 | 0.2534 | -133.506 |
| C | -14.790 | 0.1879 | -68.070 |
| H | -10.671 | -0.7897 | -70.680 |
| H | -24.518 | 0.0185 | -63.356 |
| C | -0.5597 | 0.9146 | -58.174 |
| H | 0.4474 | 0.9858 | -62.397 |
| H | -0.9250 | 19.354 | -56.707 |
| C | -0.4953 | 0.2016 | -44.640 |
| H | -0.2177 | -0.8474 | -46.112 |
| H | -14.850 | 0.2197 | -39.951 |
| C | 0.5219 | 0.8721 | -35.421 |
| H | 15.168 | 0.8467 | -39.898 |
| H | 0.5637 | 0.3551 | -25.830 |
| H | 0.2490 | 19.131 | -33.631 |
| C | -0.1632 | 14.495 | -91.187 |
| H | -0.3293 | 20.577 | -100.133 |
| H | 0.4469 | 20.633 | -84.488 |
| C | 0.6085 | 0.1810 | -94.945 |
| H | 0.0481 | -0.3947 | -102.365 |
| H | 0.7395 | -0.4518 | -86.116 |
| C | 19.835 | 0.5463 | -100.643 |
| H | 18.510 | 11.679 | -109.554 |
| H | 25.351 | 11.386 | -93.249 |
| C | 27.870 | -0.7008 | -104.251 |
| H | 22.555 | -12.969 | -111.671 |
| H | 29.545 | -13.173 | -95.404 |
| H | 37.549 | -0.4157 | -108.370 |
| C | -49.218 | 47.655 | -52.751 |
| H | -59.405 | 45.463 | -56.077 |
| H | -49.748 | 56.797 | -46.788 |
| C | -44.623 | 35.891 | -44.040 |
| H | -45.515 | 26.718 | -49.920 |
| H | -51.461 | 35.065 | -35.518 |
| C | -30.235 | 36.924 | -38.923 |
| H | -23.502 | 38.733 | -47.362 |
| H | -27.428 | 27.268 | -34.576 |
| C | -28.386 | 47.819 | -28.388 |
| H | -30.783 | 57.628 | -32.491 |
| H | -18.047 | 47.988 | -24.920 |
| H | -34.887 | 45.989 | -19.812 |
| C | -50.810 | 58.038 | -79.846 |
| H | -45.023 | 63.274 | -87.516 |
| H | -57.106 | 65.471 | -74.867 |
| C | -59.554 | 47.374 | -86.594 |
| H | -53.098 | 40.323 | -91.909 |
| H | -64.984 | 41.675 | -78.982 |
| C | -69.459 | 53.679 | -96.413 |
| H | -63.958 | 59.522 | -103.853 |
| H | -76.089 | 60.520 | -91.024 |
| C | -77.741 | 42.948 | -103.448 |
| H | -71.260 | 36.172 | -109.029 |
| H | -84.723 | 47.593 | -110.404 |
| H | -83.419 | 37.110 | -96.183 |
| H | -21.408 | 66.133 | -72.581 |
| C | -27.466 | 64.303 | -63.647 |
| H | -20.537 | 60.596 | -56.032 |
| C | -33.711 | 77.494 | -58.970 |
| H | -40.342 | 75.770 | -50.437 |
| H | -39.712 | 81.807 | -67.031 |
| C | -22.756 | 87.406 | -54.883 |
| H | -17.032 | 83.217 | -46.522 |
| H | -15.839 | 88.817 | -63.256 |
| C | -28.679 | 100.879 | -50.817 |
| H | -20.742 | 107.665 | -47.694 |
| H | -35.650 | 99.647 | -42.521 |
| H | -34.019 | 105.390 | -59.181 |

## (Δ,Δ,Λ,Λ)$S_4$-4

| | x-coord. | y-coord. | z-coord. |
|---|---|---|---|
| Fe | -0.487 | -1.364 | -12.441 |
| Fe | -10.907 | -4.987 | 4.893 |
| N | -1.836 | -2.368 | -13.458 |
| C | -3.681 | -4.094 | -14.658 |
| C | -2.703 | -1.923 | -14.372 |
| C | -1.873 | -3.680 | -13.136 |
| C | -2.785 | -4.563 | -13.716 |
| C | -3.630 | -2.747 | -14.990 |
| H | -2.662 | -0.873 | -14.632 |
| H | -2.771 | -5.605 | -13.421 |
| H | -4.397 | -4.759 | -15.126 |
| N | 0.965 | -1.749 | -13.679 |
| C | 3.280 | -2.133 | -15.186 |
| C | 2.112 | -1.106 | -13.362 |
| C | 0.967 | -2.569 | -14.731 |
| C | 2.097 | -2.786 | -15.504 |
| C | 3.283 | -1.278 | -14.098 |
| H | 0.036 | -3.071 | -14.971 |
| H | 2.041 | -3.462 | -16.347 |
| H | 4.176 | -0.739 | -13.808 |
| H | 4.176 | -2.285 | -15.776 |
| N | -0.995 | 0.282 | -13.348 |
| C | -2.020 | 2.652 | -14.402 |
| C | -2.023 | 0.919 | -12.745 |
| C | -0.480 | 0.815 | -14.458 |
| C | -0.962 | 1.991 | -15.011 |
| C | -2.556 | 2.104 | -13.250 |
| H | 0.342 | 0.289 | -14.928 |
| H | -3.381 | 2.577 | -12.733 |
| H | -2.417 | 3.570 | -14.818 |
| N | -11.015 | -5.987 | 6.569 |
| C | -11.003 | -7.088 | 9.136 |
| C | -11.609 | -7.157 | 6.817 |
| C | -10.400 | -5.355 | 7.595 |
| C | -10.381 | -5.878 | 8.886 |
| C | -11.624 | -7.735 | 8.077 |
| H | -12.095 | -7.654 | 5.985 |
| H | -9.878 | -5.330 | 9.673 |
| H | -12.124 | -8.685 | 8.218 |
| H | -11.006 | -7.518 | 10.131 |
| N | -11.969 | -6.337 | 3.919 |
| C | -13.115 | -8.408 | 2.432 |
| C | -13.293 | -6.431 | 3.778 |
| C | -11.200 | -7.280 | 3.331 |
| C | -11.742 | -8.322 | 2.581 |
| C | -13.899 | -7.443 | 3.049 |
| H | -13.902 | -5.679 | 4.266 |
| H | -11.080 | -9.051 | 2.130 |
| H | -14.978 | -7.468 | 2.974 |
| H | -13.564 | -9.208 | 1.857 |
| N | -12.514 | -3.939 | 5.265 |
| C | -14.877 | -2.457 | 5.352 |
| C | -12.920 | -3.216 | 4.195 |
| C | -13.265 | -3.914 | 6.367 |
| C | -14.444 | -3.189 | 6.449 |
| C | -14.097 | -2.471 | 4.209 |
| H | -12.920 | -4.492 | 7.217 |
| H | -15.013 | -3.205 | 7.370 |
| H | -14.383 | -1.914 | 3.325 |
| H | -15.798 | -1.889 | 5.390 |
| C | 1.990 | -0.229 | -12.231 |
| H | 2.818 | 0.415 | -11.941 |
| C | -0.887 | -4.090 | -12.182 |
| H | -0.853 | -5.137 | -11.896 |
| C | -2.495 | 0.274 | -11.554 |
| H | -3.328 | 0.709 | -11.005 |
| C | -9.793 | -7.124 | 3.571 |
| H | -9.094 | -7.852 | 3.164 |
| C | -9.782 | -4.112 | 7.220 |
| H | -9.274 | -3.505 | 7.966 |
| C | -12.001 | -3.262 | 3.089 |
| H | -12.192 | -2.692 | 2.183 |
| N | -10.916 | -3.941 | 3.256 |
| N | -9.396 | -6.135 | 4.302 |
| N | -9.863 | -3.743 | 5.985 |
| N | -0.063 | -3.209 | -11.697 |
| N | 0.872 | -0.236 | -11.582 |
| N | -1.913 | -0.815 | -11.165 |
| C | -2.322 | -1.324 | -9.912 |
| C | -3.134 | -2.271 | -7.389 |
| C | -2.496 | -2.692 | -9.690 |
| C | -2.559 | -0.431 | -8.856 |
| C | -2.944 | -0.898 | -7.617 |
| C | -2.902 | -3.156 | -8.454 |
| H | -2.352 | -3.394 | -10.500 |
| H | -2.398 | 0.631 | -9.001 |
| H | -3.109 | -0.197 | -6.807 |
| H | -3.055 | -4.219 | -8.304 |

| | | | | | | | |
|---|---|---|---|---|---|---|---|
| C | 0.720 | 0.751 | -10.575 | H | -7.632 | -6.272 | 2.342 |
| C | 0.497 | 2.745 | -8.606 | H | -5.195 | -6.024 | 2.533 |
| C | 0.317 | 0.421 | -9.281 | H | -5.587 | -5.488 | 6.774 |
| C | 0.991 | 2.089 | -10.886 | C | -3.811 | -5.536 | 4.761 |
| C | 0.873 | 3.069 | -9.920 | C | -2.606 | -5.364 | 4.815 |
| C | 0.223 | 1.401 | -8.311 | C | -7.275 | 1.177 | 5.061 |
| H | 0.098 | -0.605 | -9.019 | C | -6.743 | 2.267 | 4.948 |
| H | 1.267 | 2.352 | -11.901 | C | -7.151 | -3.818 | -0.929 |
| H | 1.076 | 4.103 | -10.176 | C | -6.317 | -3.710 | -1.810 |
| H | -0.068 | 1.126 | -7.303 | C | 0.389 | 3.726 | -7.589 |
| C | 0.963 | -3.680 | -10.863 | C | 0.288 | 4.536 | -6.686 |
| C | 2.858 | -4.616 | -8.988 | H | -0.509 | 2.377 | -15.916 |
| C | 1.389 | -5.022 | -10.874 | H | -4.303 | -2.327 | -15.726 |
| C | 1.596 | -2.803 | -9.978 | C | 3.684 | -5.078 | -7.941 |
| C | 2.523 | -3.253 | -9.065 | C | 4.321 | -5.434 | -6.964 |
| C | 2.303 | -5.479 | -9.950 | C | -3.604 | -2.716 | -6.131 |
| H | 1.017 | -5.714 | -11.620 | C | -4.081 | -3.037 | -5.057 |
| H | 1.338 | -1.757 | -9.991 | Fe | -0.108 | 12.371 | 2.770 |
| H | 2.992 | -2.549 | -8.387 | Fe | 9.665 | -5.453 | 4.141 |
| H | 2.598 | -6.521 | -9.960 | N | 0.947 | 13.220 | 4.176 |
| C | -9.201 | -2.531 | 5.661 | C | 2.263 | 14.185 | 6.440 |
| C | -7.911 | -0.079 | 5.227 | C | 1.741 | 14.290 | 4.102 |
| C | -9.888 | -1.474 | 5.063 | C | 0.806 | 12.609 | 5.375 |
| C | -7.856 | -2.370 | 6.012 | C | 1.447 | 13.070 | 6.522 |
| C | -7.219 | -1.165 | 5.787 | C | 2.410 | 14.798 | 5.204 |
| C | -9.253 | -0.264 | 4.858 | H | 1.847 | 14.764 | 3.133 |
| H | -10.934 | -1.588 | 4.805 | H | 1.298 | 12.549 | 7.460 |
| H | -7.313 | -3.202 | 6.444 | H | 2.772 | 14.567 | 7.317 |
| H | -6.178 | -1.048 | 6.065 | N | -1.706 | 13.427 | 3.171 |
| H | -9.803 | 0.562 | 4.424 | C | -4.097 | 14.856 | 3.341 |
| C | -9.981 | -3.951 | 2.198 | C | -2.695 | 13.235 | 2.268 |
| C | -8.109 | -3.892 | 0.113 | C | -1.907 | 14.309 | 4.151 |
| C | -8.649 | -3.636 | 2.464 | C | -3.082 | 15.036 | 4.270 |
| C | -10.377 | -4.249 | 0.891 | C | -3.897 | 13.937 | 2.325 |
| C | -9.451 | -4.225 | -0.135 | H | -1.107 | 14.445 | 4.870 |
| C | -7.733 | -3.604 | 1.433 | H | -3.190 | 15.738 | 5.087 |
| H | -8.342 | -3.393 | 3.473 | H | -4.656 | 13.753 | 1.575 |
| H | -11.407 | -4.525 | 0.694 | H | -5.022 | 15.417 | 3.410 |
| H | -9.763 | -4.464 | -1.145 | N | 0.467 | 13.661 | 1.399 |
| H | -6.702 | -3.361 | 1.645 | C | 1.520 | 15.179 | -0.695 |
| C | -7.996 | -5.998 | 4.446 | C | 1.526 | 13.220 | 0.686 |
| C | -5.213 | -5.708 | 4.665 | C | -0.055 | 14.849 | 1.082 |
| C | -7.408 | -5.776 | 5.692 | C | 0.440 | 15.631 | 0.051 |
| C | -7.181 | -6.108 | 3.313 | C | 2.071 | 13.954 | -0.367 |
| C | -5.814 | -5.955 | 3.420 | H | -0.895 | 15.197 | 1.671 |
| C | -6.036 | -5.642 | 5.800 | H | 2.920 | 13.555 | -0.907 |
| H | -8.023 | -5.751 | 6.582 | H | 1.924 | 15.772 | -1.507 |

| | | | |
|---|---|---|---|
| N | 10.768 | -4.498 | 5.426 |
| C | 12.008 | -2.860 | 7.308 |
| C | 12.098 | -4.402 | 5.476 |
| C | 10.035 | -3.791 | 6.313 |
| C | 10.629 | -2.960 | 7.263 |
| C | 12.750 | -3.602 | 6.399 |
| H | 12.665 | -4.985 | 4.758 |
| H | 10.001 | -2.410 | 7.953 |
| H | 13.832 | -3.565 | 6.400 |
| H | 12.495 | -2.224 | 8.037 |
| N | 11.167 | -5.990 | 3.034 |
| C | 13.165 | -6.502 | 1.161 |
| C | 11.836 | -7.145 | 3.049 |
| C | 11.481 | -5.072 | 2.091 |
| C | 12.476 | -5.302 | 1.143 |
| C | 12.837 | -7.434 | 2.136 |
| H | 11.568 | -7.862 | 3.817 |
| H | 12.697 | -4.537 | 0.409 |
| H | 13.352 | -8.384 | 2.196 |
| H | 13.945 | -6.705 | 0.436 |
| N | 9.620 | -7.075 | 5.217 |
| C | 9.125 | -9.449 | 6.595 |
| C | 8.797 | -8.015 | 4.702 |
| C | 10.197 | -7.314 | 6.397 |
| C | 9.978 | -8.482 | 7.109 |
| C | 8.528 | -9.209 | 5.371 |
| H | 10.861 | -6.552 | 6.789 |
| H | 10.478 | -8.626 | 8.059 |
| H | 7.862 | -9.933 | 4.917 |
| H | 8.936 | -10.369 | 7.135 |
| C | -2.389 | 12.234 | 1.281 |
| H | -3.109 | 11.989 | 0.503 |
| C | -0.063 | 11.463 | 5.335 |
| H | -0.272 | 10.895 | 6.239 |
| C | 2.058 | 11.968 | 1.144 |
| H | 2.949 | 11.557 | 0.673 |
| C | 10.740 | -3.848 | 2.209 |
| H | 10.985 | -2.995 | 1.580 |
| C | 8.621 | -4.005 | 6.211 |
| H | 7.955 | -3.491 | 6.900 |
| C | 8.292 | -7.696 | 3.399 |
| H | 7.683 | -8.423 | 2.867 |
| N | 8.609 | -6.552 | 2.876 |
| N | 9.808 | -3.793 | 3.105 |
| N | 8.179 | -4.817 | 5.301 |
| N | -0.592 | 11.153 | 4.200 |
| N | -1.262 | 11.615 | 1.390 |
| N | 1.494 | 11.389 | 2.153 |
| C | 2.073 | 10.163 | 2.557 |
| C | 3.375 | 7.783 | 3.289 |
| C | 2.398 | 9.918 | 3.893 |
| C | 2.389 | 9.202 | 1.589 |
| C | 3.010 | 8.027 | 1.954 |
| C | 3.048 | 8.752 | 4.250 |
| H | 2.196 | 10.671 | 4.644 |
| H | 2.120 | 9.375 | 0.554 |
| H | 3.236 | 7.285 | 1.196 |
| H | 3.329 | 8.591 | 5.284 |
| C | -0.963 | 10.662 | 0.388 |
| C | -0.467 | 8.773 | -1.623 |
| C | -0.576 | 9.366 | 0.729 |
| C | -1.080 | 11.021 | -0.958 |
| C | -0.831 | 10.090 | -1.947 |
| C | -0.333 | 8.436 | -0.267 |
| H | -0.495 | 9.086 | 1.773 |
| H | -1.342 | 12.041 | -1.218 |
| H | -0.921 | 10.374 | -2.989 |
| H | -0.032 | 7.430 | 0.003 |
| C | -1.431 | 10.010 | 4.195 |
| C | -3.124 | 7.790 | 4.404 |
| C | -0.923 | 8.781 | 4.623 |
| C | -2.778 | 10.122 | 3.845 |
| C | -3.613 | 9.029 | 3.959 |
| C | -1.758 | 7.684 | 4.714 |
| H | 0.130 | 8.695 | 4.867 |
| H | -3.177 | 11.079 | 3.535 |
| H | -4.664 | 9.129 | 3.717 |
| H | -1.359 | 6.731 | 5.041 |
| C | 6.800 | -5.103 | 5.316 |
| C | 3.996 | -5.385 | 5.313 |
| C | 6.080 | -5.120 | 6.522 |
| C | 6.106 | -5.341 | 4.125 |
| C | 4.735 | -5.482 | 4.123 |
| C | 4.706 | -5.251 | 6.517 |
| H | 6.604 | -5.045 | 7.467 |
| H | 6.635 | -5.375 | 3.185 |
| H | 4.216 | -5.646 | 3.186 |
| H | 4.165 | -5.239 | 7.456 |
| C | 8.278 | -6.374 | 1.516 |
| C | 7.381 | -6.147 | -1.144 |
| C | 7.842 | -5.142 | 1.017 |
| C | 8.362 | -7.465 | 0.636 |
| C | 7.922 | -7.352 | -0.666 |
| C | 7.390 | -5.036 | -0.283 |

| | | | | | | | |
|---|---|---|---|---|---|---|---|
| H | 7.820 | -4.272 | 1.661 | H | -5.224 | -0.697 | -5.095 |
| H | 8.794 | -8.399 | 0.977 | H | -6.595 | 0.047 | -4.304 |
| H | 7.983 | -8.207 | -1.329 | C | -6.806 | -0.162 | -6.443 |
| H | 7.019 | -4.083 | -0.642 | H | -6.511 | -0.868 | -7.225 |
| C | 9.258 | -2.526 | 3.393 | H | -7.896 | -0.083 | -6.451 |
| C | 8.210 | 0.017 | 3.992 | C | -6.196 | 1.210 | -6.748 |
| C | 7.888 | -2.357 | 3.606 | H | -5.105 | 1.150 | -6.662 |
| C | 10.106 | -1.416 | 3.517 | H | -6.548 | 1.936 | -6.007 |
| C | 9.590 | -0.168 | 3.811 | C | -6.571 | 1.684 | -8.150 |
| C | 7.376 | -1.110 | 3.896 | H | -6.198 | 0.986 | -8.901 |
| H | 7.218 | -3.201 | 3.515 | H | -6.138 | 2.666 | -8.342 |
| H | 11.176 | -1.548 | 3.411 | H | -7.654 | 1.757 | -8.252 |
| H | 10.259 | 0.680 | 3.904 | C | -7.238 | -3.355 | -6.023 |
| H | 6.309 | -0.991 | 4.046 | H | -6.412 | -3.210 | -6.725 |
| C | 7.628 | 1.290 | 4.200 | H | -7.170 | -4.396 | -5.693 |
| C | 7.025 | 2.348 | 4.271 | C | -8.581 | -3.143 | -6.734 |
| C | 2.583 | -5.386 | 5.265 | H | -8.702 | -2.091 | -7.001 |
| C | 1.374 | -5.298 | 5.143 | H | -9.399 | -3.414 | -6.061 |
| C | 6.814 | -6.067 | -2.438 | C | -8.677 | -3.982 | -8.016 |
| C | 6.266 | -6.040 | -3.527 | H | -7.811 | -3.771 | -8.652 |
| C | -0.256 | 7.820 | -2.653 | H | -9.572 | -3.665 | -8.559 |
| C | -0.114 | 7.039 | -3.576 | C | -8.765 | -5.482 | -7.744 |
| H | -0.022 | 16.588 | -0.157 | H | -7.853 | -5.852 | -7.274 |
| H | 3.040 | 15.672 | 5.086 | H | -8.908 | -6.017 | -8.683 |
| C | -3.984 | 6.671 | 4.525 | H | -9.609 | -5.703 | -7.089 |
| C | -4.717 | 5.698 | 4.576 | C | -8.432 | -2.127 | -3.604 |
| C | 4.094 | 6.609 | 3.624 | H | -8.948 | -3.093 | -3.597 |
| C | 4.761 | 5.622 | 3.879 | H | -8.149 | -1.931 | -2.565 |
| Pt | -5.137 | -3.414 | -3.397 | C | -9.396 | -1.028 | -4.064 |
| Pt | 5.296 | -5.795 | -5.263 | H | -9.719 | -1.205 | -5.092 |
| Pt | 0.106 | 5.788 | -5.136 | H | -8.893 | -0.057 | -4.033 |
| Pt | 5.903 | 3.997 | 4.114 | C | -10.624 | -0.987 | -3.147 |
| Pt | -0.613 | -5.260 | 4.954 | H | -11.131 | -1.957 | -3.179 |
| Pt | -5.767 | 4.007 | 4.724 | H | -10.298 | -0.817 | -2.114 |
| P | 0.051 | 7.648 | -6.481 | C | -11.593 | 0.114 | -3.569 |
| P | 0.252 | 4.071 | -3.644 | H | -11.938 | -0.051 | -4.590 |
| P | 6.270 | -7.687 | -6.135 | H | -11.108 | 1.090 | -3.520 |
| P | 4.468 | -3.839 | -4.406 | H | -12.459 | 0.123 | -2.907 |
| P | -6.844 | -2.358 | -4.520 | C | -3.429 | -5.282 | -0.866 |
| P | -3.313 | -4.506 | -2.545 | H | -4.015 | -6.200 | -0.983 |
| P | -0.808 | -7.210 | 6.155 | H | -4.034 | -4.614 | -0.246 |
| P | -0.562 | -3.278 | 3.786 | C | -2.115 | -5.597 | -0.139 |
| P | 7.259 | 5.079 | 5.618 | H | -1.630 | -4.664 | 0.165 |
| P | 4.734 | 3.032 | 2.391 | H | -1.424 | -6.121 | -0.805 |
| P | -6.266 | 4.445 | 6.917 | C | -2.359 | -6.466 | 1.099 |
| P | -5.298 | 3.179 | 2.627 | H | -2.899 | -7.373 | 0.807 |
| C | -6.317 | -0.670 | -5.082 | H | -2.985 | -5.921 | 1.813 |

| | | | |
|---|---|---|---|
| C | -1.043 | -6.852 | 1.767 |
| H | -0.492 | -5.963 | 2.076 |
| H | -0.417 | -7.427 | 1.084 |
| H | -1.233 | -7.455 | 2.653 |
| C | -2.945 | -5.881 | -3.727 |
| H | -2.721 | -5.395 | -4.682 |
| H | -3.919 | -6.362 | -3.866 |
| C | -1.912 | -6.983 | -3.454 |
| H | -2.135 | -7.805 | -4.142 |
| H | -2.027 | -7.375 | -2.440 |
| C | -0.464 | -6.542 | -3.687 |
| H | -0.169 | -5.800 | -2.938 |
| H | -0.386 | -6.070 | -4.672 |
| C | 0.497 | -7.727 | -3.615 |
| H | 1.519 | -7.389 | -3.790 |
| H | 0.244 | -8.472 | -4.369 |
| H | 0.451 | -8.198 | -2.632 |
| C | -1.769 | -3.476 | -2.500 |
| H | -0.913 | -4.073 | -2.178 |
| H | -1.578 | -3.167 | -3.532 |
| C | -1.848 | -2.226 | -1.614 |
| H | -0.837 | -1.812 | -1.522 |
| H | -2.180 | -2.498 | -0.606 |
| C | -2.778 | -1.154 | -2.185 |
| H | -2.516 | -0.962 | -3.230 |
| H | -3.808 | -1.516 | -2.170 |
| C | -2.691 | 0.143 | -1.388 |
| H | -3.359 | 0.889 | -1.817 |
| H | -1.676 | 0.539 | -1.405 |
| H | -2.978 | -0.023 | -0.348 |
| C | -4.339 | 1.603 | 2.828 |
| H | -4.255 | 1.101 | 1.861 |
| H | -4.918 | 0.956 | 3.491 |
| C | -2.925 | 1.771 | 3.399 |
| H | -2.362 | 2.488 | 2.793 |
| H | -2.417 | 0.803 | 3.315 |
| C | -2.902 | 2.213 | 4.862 |
| H | -3.544 | 1.554 | 5.454 |
| H | -3.308 | 3.222 | 4.945 |
| C | -1.485 | 2.201 | 5.428 |
| H | -1.496 | 2.522 | 6.470 |
| H | -0.842 | 2.877 | 4.862 |
| H | -1.059 | 1.198 | 5.380 |
| C | -4.334 | 4.011 | 1.257 |
| H | -5.030 | 4.410 | 0.514 |
| H | -3.716 | 3.267 | 0.745 |
| C | -3.454 | 5.154 | 1.778 |
| H | -4.095 | 6.015 | 1.982 |
| H | -2.993 | 4.865 | 2.727 |
| C | -2.355 | 5.566 | 0.795 |
| H | -2.034 | 6.583 | 1.043 |
| H | -2.752 | 5.586 | -0.225 |
| C | -1.144 | 4.637 | 0.863 |
| H | -0.367 | 4.973 | 0.175 |
| H | -1.423 | 3.617 | 0.594 |
| H | -0.729 | 4.627 | 1.872 |
| C | -6.880 | 2.665 | 1.802 |
| H | -7.232 | 3.519 | 1.214 |
| H | -7.591 | 2.550 | 2.624 |
| C | -6.951 | 1.394 | 0.949 |
| H | -8.010 | 1.202 | 0.741 |
| H | -6.576 | 0.540 | 1.520 |
| C | -6.204 | 1.499 | -0.381 |
| H | -5.129 | 1.598 | -0.198 |
| H | -6.533 | 2.398 | -0.913 |
| C | -6.458 | 0.273 | -1.255 |
| H | -5.900 | 0.356 | -2.188 |
| H | -7.519 | 0.188 | -1.491 |
| H | -6.143 | -0.640 | -0.748 |
| C | -5.376 | 3.162 | 7.908 |
| H | -4.421 | 3.013 | 7.394 |
| H | -5.932 | 2.228 | 7.784 |
| C | -5.095 | 3.415 | 9.393 |
| H | -4.481 | 4.313 | 9.504 |
| H | -6.028 | 3.576 | 9.938 |
| C | -4.356 | 2.218 | 10.001 |
| H | -3.418 | 2.058 | 9.457 |
| H | -4.966 | 1.316 | 9.881 |
| C | -4.061 | 2.444 | 11.482 |
| H | -3.438 | 3.329 | 11.616 |
| H | -3.535 | 1.583 | 11.894 |
| H | -4.988 | 2.585 | 12.038 |
| C | -5.741 | 6.099 | 7.547 |
| H | -4.653 | 6.069 | 7.663 |
| H | -5.938 | 6.801 | 6.731 |
| C | -6.390 | 6.638 | 8.829 |
| H | -6.232 | 5.945 | 9.659 |
| H | -7.468 | 6.742 | 8.678 |
| C | -5.796 | 8.004 | 9.191 |
| H | -4.715 | 7.900 | 9.340 |
| H | -5.952 | 8.698 | 8.359 |
| C | -6.439 | 8.563 | 10.458 |
| H | -6.002 | 9.532 | 10.701 |
| H | -6.276 | 7.889 | 11.299 |

| | | | | | | | |
|---|---|---|---|---|---|---|---|
| H | -7.512 | 8.691 | 10.317 | H | 10.608 | 6.975 | 3.273 |
| C | -8.070 | 4.266 | 7.271 | H | 11.495 | 5.858 | 4.320 |
| H | -8.551 | 5.215 | 7.012 | C | 11.815 | 7.942 | 4.779 |
| H | -8.439 | 3.530 | 6.550 | H | 12.155 | 7.765 | 5.800 |
| C | -8.502 | 3.833 | 8.678 | H | 11.268 | 8.885 | 4.753 |
| H | -8.136 | 4.541 | 9.426 | H | 12.687 | 8.028 | 4.132 |
| H | -8.075 | 2.852 | 8.906 | C | 5.624 | 1.576 | 1.686 |
| C | -10.030 | 3.753 | 8.763 | H | 6.673 | 1.888 | 1.688 |
| H | -10.457 | 4.734 | 8.529 | H | 5.553 | 0.776 | 2.430 |
| H | -10.397 | 3.042 | 8.015 | C | 5.295 | 1.007 | 0.305 |
| C | -10.481 | 3.317 | 10.155 | H | 4.347 | 0.461 | 0.333 |
| H | -10.137 | 4.027 | 10.908 | H | 5.198 | 1.805 | -0.437 |
| H | -10.079 | 2.332 | 10.397 | C | 6.419 | 0.052 | -0.113 |
| H | -11.569 | 3.269 | 10.196 | H | 6.634 | -0.637 | 0.711 |
| C | 6.560 | 6.664 | 6.259 | H | 7.330 | 0.627 | -0.307 |
| H | 5.496 | 6.471 | 6.428 | C | 6.046 | -0.751 | -1.353 |
| H | 6.607 | 7.384 | 5.435 | H | 5.146 | -1.340 | -1.169 |
| C | 7.156 | 7.285 | 7.528 | H | 6.856 | -1.433 | -1.608 |
| H | 8.227 | 7.462 | 7.402 | H | 5.863 | -0.090 | -2.201 |
| H | 7.026 | 6.599 | 8.370 | C | 4.541 | 4.204 | 0.978 |
| C | 6.462 | 8.615 | 7.843 | H | 3.929 | 5.033 | 1.345 |
| H | 5.388 | 8.440 | 7.978 | H | 4.021 | 3.768 | 0.121 |
| H | 6.583 | 9.296 | 6.994 | C | 5.917 | 4.742 | 0.558 |
| C | 7.043 | 9.254 | 9.102 | H | 6.514 | 4.944 | 1.452 |
| H | 8.107 | 9.452 | 8.973 | H | 6.446 | 3.980 | -0.022 |
| H | 6.914 | 8.595 | 9.961 | C | 5.794 | 6.028 | -0.262 |
| H | 6.537 | 10.197 | 9.309 | H | 5.326 | 6.803 | 0.354 |
| C | 7.740 | 4.053 | 7.078 | H | 5.140 | 5.850 | -1.122 |
| H | 7.757 | 3.020 | 6.716 | C | 7.159 | 6.513 | -0.743 |
| H | 6.924 | 4.110 | 7.805 | H | 7.815 | 6.706 | 0.106 |
| C | 9.075 | 4.351 | 7.774 | H | 7.048 | 7.435 | -1.314 |
| H | 9.896 | 4.223 | 7.062 | H | 7.628 | 5.763 | -1.381 |
| H | 9.098 | 5.383 | 8.131 | C | 3.015 | 2.477 | 2.802 |
| C | 9.282 | 3.403 | 8.960 | H | 3.109 | 1.740 | 3.606 |
| H | 9.262 | 2.367 | 8.603 | H | 2.513 | 3.346 | 3.240 |
| H | 8.459 | 3.527 | 9.671 | C | 2.149 | 1.898 | 1.680 |
| C | 10.611 | 3.682 | 9.660 | H | 2.637 | 1.022 | 1.247 |
| H | 11.443 | 3.545 | 8.969 | H | 2.019 | 2.637 | 0.883 |
| H | 10.739 | 3.000 | 10.500 | C | 0.776 | 1.479 | 2.217 |
| H | 10.636 | 4.705 | 10.036 | H | 0.249 | 2.358 | 2.600 |
| C | 8.811 | 5.491 | 4.701 | H | 0.909 | 0.782 | 3.051 |
| H | 8.497 | 5.709 | 3.676 | C | -0.059 | 0.812 | 1.128 |
| H | 9.401 | 4.571 | 4.646 | H | 0.437 | -0.089 | 0.764 |
| C | 9.689 | 6.647 | 5.198 | H | -0.203 | 1.492 | 0.286 |
| H | 10.008 | 6.470 | 6.228 | H | -1.038 | 0.529 | 1.516 |
| H | 9.116 | 7.578 | 5.178 | C | 1.482 | 8.727 | -6.027 |
| C | 10.927 | 6.794 | 4.305 | H | 2.360 | 8.315 | -6.532 |

| | | | | | | | |
|---|---|---|---|---|---|---|---|
| H | 1.642 | 8.561 | -4.957 | H | 5.443 | 5.514 | -3.405 |
| C | 1.384 | 10.237 | -6.277 | H | 4.906 | 6.561 | -4.726 |
| H | 0.542 | 10.639 | -5.706 | H | 4.850 | 4.801 | -4.912 |
| H | 1.200 | 10.441 | -7.334 | C | 0.517 | 2.370 | -4.324 |
| C | 2.663 | 10.963 | -5.838 | H | 1.436 | 2.394 | -4.917 |
| H | 2.906 | 10.682 | -4.808 | H | -0.290 | 2.220 | -5.048 |
| H | 2.461 | 12.039 | -5.849 | C | 0.549 | 1.190 | -3.337 |
| C | 3.854 | 10.672 | -6.749 | H | -0.043 | 0.369 | -3.755 |
| H | 4.710 | 11.271 | -6.439 | H | 0.082 | 1.468 | -2.387 |
| H | 3.613 | 10.922 | -7.783 | C | 1.966 | 0.684 | -3.060 |
| H | 4.139 | 9.620 | -6.703 | H | 2.442 | 0.394 | -4.003 |
| C | -1.491 | 8.624 | -6.197 | H | 2.568 | 1.483 | -2.617 |
| H | -1.382 | 9.110 | -5.221 | C | 1.948 | -0.512 | -2.112 |
| H | -2.291 | 7.886 | -6.077 | H | 2.966 | -0.838 | -1.900 |
| C | -1.925 | 9.652 | -7.249 | H | 1.398 | -1.345 | -2.552 |
| H | -1.134 | 10.387 | -7.415 | H | 1.472 | -0.245 | -1.168 |
| H | -2.116 | 9.146 | -8.199 | C | -1.291 | 3.927 | -2.637 |
| C | -3.198 | 10.375 | -6.795 | H | -1.452 | 4.917 | -2.200 |
| H | -3.008 | 10.873 | -5.838 | H | -1.201 | 3.215 | -1.812 |
| H | -3.994 | 9.640 | -6.635 | C | -2.497 | 3.572 | -3.516 |
| C | -3.646 | 11.404 | -7.830 | H | -2.493 | 4.207 | -4.407 |
| H | -2.870 | 12.154 | -7.984 | H | -2.414 | 2.533 | -3.850 |
| H | -4.551 | 11.905 | -7.489 | C | -3.812 | 3.765 | -2.757 |
| H | -3.855 | 10.919 | -8.784 | H | -3.927 | 4.822 | -2.495 |
| C | 0.170 | 7.325 | -8.297 | H | -3.780 | 3.195 | -1.822 |
| H | 0.803 | 6.437 | -8.393 | C | -5.006 | 3.310 | -3.593 |
| H | -0.825 | 7.025 | -8.641 | H | -5.051 | 3.866 | -4.530 |
| C | 0.727 | 8.424 | -9.210 | H | -5.934 | 3.475 | -3.046 |
| H | 1.745 | 8.678 | -8.901 | H | -4.924 | 2.246 | -3.825 |
| H | 0.118 | 9.328 | -9.138 | C | 6.592 | -8.940 | -4.814 |
| C | 0.747 | 7.944 | -10.666 | H | 7.328 | -8.503 | -4.133 |
| H | 1.350 | 7.032 | -10.738 | H | 5.654 | -8.968 | -4.249 |
| H | -0.273 | 7.693 | -10.978 | C | 6.974 | -10.389 | -5.156 |
| C | 1.319 | 9.016 | -11.591 | H | 6.301 | -10.771 | -5.929 |
| H | 2.341 | 9.263 | -11.303 | H | 6.797 | -10.987 | -4.255 |
| H | 0.716 | 9.923 | -11.543 | C | 8.423 | -10.600 | -5.603 |
| H | 1.325 | 8.657 | -12.620 | H | 8.643 | -9.984 | -6.479 |
| C | 1.556 | 4.279 | -2.341 | H | 8.527 | -11.646 | -5.908 |
| H | 1.480 | 3.482 | -1.595 | C | 9.440 | -10.308 | -4.501 |
| H | 1.341 | 5.222 | -1.829 | H | 9.421 | -9.256 | -4.210 |
| C | 2.987 | 4.323 | -2.897 | H | 10.443 | -10.544 | -4.856 |
| H | 3.683 | 4.223 | -2.057 | H | 9.233 | -10.914 | -3.619 |
| H | 3.157 | 3.469 | -3.561 | C | 7.871 | -7.272 | -6.958 |
| C | 3.292 | 5.618 | -3.653 | H | 7.634 | -6.610 | -7.797 |
| H | 3.146 | 6.473 | -2.985 | H | 8.350 | -8.160 | -7.375 |
| H | 2.589 | 5.730 | -4.481 | C | 8.843 | -6.553 | -6.015 |
| C | 4.714 | 5.623 | -4.207 | H | 8.394 | -5.612 | -5.678 |

| | | | | | | | |
|---|---|---|---|---|---|---|---|
| H | 9.020 | -7.165 | -5.125 | C | 5.704 | -2.472 | -4.605 |
| C | 10.174 | -6.277 | -6.720 | H | 5.389 | -1.576 | -4.064 |
| H | 9.999 | -5.629 | -7.585 | H | 5.725 | -2.217 | -5.669 |
| H | 10.589 | -7.220 | -7.088 | C | 7.114 | -2.891 | -4.172 |
| C | 11.176 | -5.616 | -5.777 | H | 7.419 | -3.770 | -4.746 |
| H | 10.788 | -4.662 | -5.414 | H | 7.104 | -3.184 | -3.118 |
| H | 12.114 | -5.432 | -6.299 | C | 8.121 | -1.759 | -4.390 |
| H | 11.375 | -6.258 | -4.918 | H | 7.756 | -0.845 | -3.909 |
| C | 5.263 | -8.541 | -7.429 | H | 8.211 | -1.555 | -5.461 |
| H | 4.674 | -7.759 | -7.921 | C | 9.490 | -2.122 | -3.819 |
| H | 4.544 | -9.188 | -6.917 | H | 9.872 | -3.026 | -4.293 |
| C | 6.010 | -9.343 | -8.504 | H | 9.421 | -2.298 | -2.744 |
| H | 6.649 | -8.671 | -9.084 | H | 10.197 | -1.312 | -3.995 |
| H | 6.653 | -10.097 | -8.043 | C | -1.750 | -6.922 | 7.714 |
| C | 5.012 | -10.032 | -9.441 | H | -1.887 | -7.851 | 8.272 |
| H | 4.369 | -9.274 | -9.903 | H | -2.742 | -6.559 | 7.428 |
| H | 4.371 | -10.701 | -8.859 | C | -1.065 | -5.869 | 8.597 |
| C | 5.734 | -10.827 | -10.527 | H | -0.876 | -4.975 | 7.996 |
| H | 6.363 | -10.170 | -11.129 | H | -0.095 | -6.246 | 8.936 |
| H | 5.008 | -11.309 | -11.180 | C | -1.921 | -5.489 | 9.810 |
| H | 6.364 | -11.597 | -10.080 | H | -1.445 | -4.638 | 10.308 |
| C | 2.989 | -3.019 | -5.175 | H | -2.909 | -5.163 | 9.467 |
| H | 3.240 | -2.893 | -6.232 | C | -2.071 | -6.631 | 10.813 |
| H | 2.853 | -2.019 | -4.754 | H | -1.092 | -6.988 | 11.134 |
| C | 1.689 | -3.824 | -5.064 | H | -2.620 | -7.467 | 10.377 |
| H | 1.899 | -4.875 | -5.288 | H | -2.617 | -6.283 | 11.689 |
| H | 1.311 | -3.769 | -4.038 | C | -1.747 | -8.523 | 5.250 |
| C | 0.603 | -3.331 | -6.028 | H | -1.032 | -9.087 | 4.644 |
| H | 0.955 | -3.437 | -7.059 | H | -2.404 | -7.999 | 4.551 |
| H | -0.276 | -3.973 | -5.911 | C | -2.598 | -9.493 | 6.081 |
| C | 0.194 | -1.880 | -5.782 | H | -1.978 | -10.016 | 6.814 |
| H | 1.024 | -1.199 | -5.974 | H | -3.363 | -8.934 | 6.627 |
| H | -0.137 | -1.743 | -4.751 | C | -3.276 | -10.518 | 5.165 |
| H | -0.629 | -1.609 | -6.444 | H | -2.511 | -11.077 | 4.617 |
| C | 4.091 | -3.840 | -2.590 | H | -3.895 | -9.991 | 4.430 |
| H | 5.045 | -3.815 | -2.056 | C | -4.142 | -11.487 | 5.968 |
| H | 3.532 | -2.946 | -2.301 | H | -3.535 | -12.032 | 6.691 |
| C | 3.329 | -5.100 | -2.162 | H | -4.614 | -12.206 | 5.299 |
| H | 3.909 | -5.981 | -2.446 | H | -4.923 | -10.947 | 6.506 |
| H | 2.370 | -5.155 | -2.685 | C | 0.782 | -7.984 | 6.677 |
| C | 3.083 | -5.106 | -0.652 | H | 1.355 | -8.179 | 5.764 |
| H | 4.043 | -5.044 | -0.128 | H | 1.339 | -7.214 | 7.221 |
| H | 2.496 | -4.222 | -0.381 | C | 0.708 | -9.260 | 7.522 |
| C | 2.339 | -6.362 | -0.206 | H | 0.244 | -10.063 | 6.942 |
| H | 2.904 | -7.257 | -0.469 | H | 0.093 | -9.088 | 8.410 |
| H | 1.361 | -6.416 | -0.686 | C | 2.112 | -9.697 | 7.956 |
| H | 2.194 | -6.348 | 0.875 | H | 2.729 | -9.864 | 7.066 |

| | | | |
|---|---|---|---|
| H | 2.577 | -8.892 | 8.536 |
| C | 2.058 | -10.972 | 8.794 |
| H | 3.065 | -11.264 | 9.092 |
| H | 1.614 | -11.787 | 8.222 |
| H | 1.462 | -10.813 | 9.693 |
| C | 0.591 | -3.116 | 2.334 |
| H | 0.436 | -2.129 | 1.884 |
| H | 0.301 | -3.851 | 1.579 |
| C | 2.075 | -3.297 | 2.688 |
| H | 2.369 | -4.327 | 2.473 |
| H | 2.236 | -3.138 | 3.758 |
| C | 2.964 | -2.325 | 1.909 |
| H | 2.745 | -1.301 | 2.229 |
| H | 2.743 | -2.390 | 0.838 |
| C | 4.442 | -2.626 | 2.145 |
| H | 4.706 | -3.601 | 1.732 |
| H | 4.661 | -2.634 | 3.213 |
| H | 5.065 | -1.871 | 1.667 |
| C | -0.119 | -1.726 | 4.720 |
| H | 0.898 | -1.444 | 4.434 |
| H | -0.771 | -0.905 | 4.407 |
| C | -0.152 | -1.859 | 6.248 |
| H | 0.300 | -0.957 | 6.674 |
| H | 0.460 | -2.713 | 6.548 |
| C | -1.568 | -2.026 | 6.802 |
| H | -1.963 | -2.997 | 6.488 |
| H | -2.218 | -1.247 | 6.388 |
| C | -1.583 | -1.934 | 8.326 |
| H | -1.268 | -0.942 | 8.652 |
| H | -0.905 | -2.670 | 8.759 |
| H | -2.588 | -2.122 | 8.703 |
| C | -2.137 | -2.871 | 2.893 |
| H | -1.930 | -2.202 | 2.052 |
| H | -2.484 | -3.821 | 2.475 |
| C | -3.256 | -2.280 | 3.755 |
| H | -3.340 | -2.844 | 4.687 |
| H | -3.010 | -1.245 | 4.010 |
| C | -4.609 | -2.322 | 3.039 |
| H | -5.319 | -1.714 | 3.609 |
| H | -4.981 | -3.352 | 3.043 |
| C | -4.543 | -1.816 | 1.598 |
| H | -4.135 | -2.578 | 0.934 |
| H | -3.916 | -0.928 | 1.527 |
| H | -5.540 | -1.557 | 1.243 |

## (Δ,Δ,Δ,Λ)-$C_3$-4

| | x-coord. | y-coord. | z-coord. |
|---|---|---|---|
| Fe | 9.139 | 6.710 | 3.008 |
| Fe | -4.369 | -6.810 | 8.912 |
| Fe | -8.249 | 7.136 | -5.533 |
| Fe | 4.181 | -8.467 | -9.119 |
| N | -3.781 | -7.229 | 10.746 |
| C | -2.685 | -8.094 | 13.164 |
| C | -4.444 | -7.092 | 11.889 |
| C | -2.552 | -7.793 | 10.792 |
| C | -1.978 | -8.239 | 11.985 |
| C | -3.942 | -7.503 | 13.113 |
| H | -5.422 | -6.639 | 11.829 |
| H | -0.998 | -8.688 | 11.972 |
| H | -4.528 | -7.367 | 14.007 |
| H | -2.272 | -8.432 | 14.101 |
| N | -4.651 | -8.731 | 8.483 |
| C | -4.828 | -11.317 | 7.457 |
| C | -4.851 | -8.939 | 7.164 |
| C | -4.576 | -9.791 | 9.281 |
| C | -4.661 | -11.095 | 8.819 |
| C | -4.933 | -10.224 | 6.619 |
| H | -4.459 | -9.596 | 10.336 |
| H | -4.600 | -11.916 | 9.514 |
| H | -5.083 | -10.344 | 5.558 |
| H | -4.889 | -12.321 | 7.068 |
| N | -6.239 | -6.560 | 9.431 |
| C | -8.789 | -5.898 | 10.361 |
| C | -7.228 | -7.449 | 9.421 |
| C | -6.510 | -5.321 | 9.897 |
| C | -7.776 | -4.960 | 10.369 |
| C | -8.507 | -7.170 | 9.872 |
| H | -6.991 | -8.431 | 9.044 |
| H | -7.943 | -3.961 | 10.738 |
| H | -9.265 | -7.935 | 9.847 |
| H | -9.774 | -5.655 | 10.726 |
| N | 9.281 | 8.608 | 2.577 |
| C | 8.846 | 11.292 | 1.967 |
| C | 10.009 | 9.564 | 3.143 |
| C | 8.354 | 8.968 | 1.665 |
| C | 8.104 | 10.306 | 1.347 |
| C | 9.833 | 10.910 | 2.870 |
| H | 10.774 | 9.243 | 3.833 |
| H | 7.339 | 10.548 | 0.626 |
| H | 10.455 | 11.644 | 3.355 |
| H | 8.676 | 12.334 | 1.748 |
| N | 10.758 | 6.229 | 2.101 |
| C | 13.137 | 5.194 | 1.078 |
| C | 11.052 | 4.915 | 2.220 |
| C | 11.628 | 7.010 | 1.471 |
| C | 12.823 | 6.543 | 0.948 |
| C | 12.235 | 4.366 | 1.717 |
| H | 11.362 | 8.051 | 1.381 |
| H | 13.495 | 7.223 | 0.452 |
| H | 12.429 | 3.312 | 1.840 |
| H | 14.064 | 4.805 | 0.688 |
| N | 10.080 | 6.985 | 4.689 |
| C | 11.152 | 7.800 | 7.137 |
| C | 11.338 | 6.708 | 5.014 |
| C | 9.329 | 7.656 | 5.591 |
| C | 9.837 | 8.082 | 6.821 |
| C | 11.914 | 7.089 | 6.215 |
| H | 11.919 | 6.161 | 4.288 |
| H | 9.201 | 8.622 | 7.503 |
| H | 12.941 | 6.837 | 6.422 |
| H | 11.577 | 8.119 | 8.074 |
| N | -10.121 | 6.606 | -5.702 |
| C | -12.653 | 5.517 | -6.155 |
| C | -11.217 | 7.352 | -5.611 |
| C | -10.273 | 5.298 | -6.010 |
| C | -11.526 | 4.723 | -6.242 |
| C | -12.493 | 6.859 | -5.828 |
| H | -11.073 | 8.391 | -5.356 |
| H | -11.596 | 3.676 | -6.489 |
| H | -13.344 | 7.514 | -5.744 |
| H | -13.634 | 5.107 | -6.334 |
| N | -8.040 | 7.476 | -7.456 |
| C | -7.538 | 8.394 | -10.044 |
| C | -6.922 | 8.184 | -7.739 |
| C | -8.880 | 7.215 | -8.452 |
| C | -8.675 | 7.647 | -9.752 |
| C | -6.645 | 8.660 | -9.024 |
| H | -9.760 | 6.641 | -8.204 |
| H | -9.394 | 7.409 | -10.518 |
| H | -5.747 | 9.229 | -9.200 |
| H | -7.358 | 8.754 | -11.044 |
| N | -8.717 | 9.046 | -5.219 |
| C | -9.135 | 11.683 | -4.404 |
| C | -9.006 | 9.999 | -6.099 |
| C | -8.662 | 9.386 | -3.913 |

| | | | | | | | |
|---|---|---|---|---|---|---|---|
| C | -8.858 | 10.699 | -3.475 | H | 10.125 | 3.092 | 3.011 |
| C | -9.223 | 11.321 | -5.744 | C | -9.042 | 4.569 | -6.062 |
| H | -9.086 | 9.698 | -7.132 | H | -9.057 | 3.505 | -6.282 |
| H | -8.797 | 10.925 | -2.423 | C | -8.444 | 8.288 | -3.023 |
| H | -9.461 | 12.049 | -6.502 | H | -8.438 | 8.475 | -1.953 |
| H | -9.293 | 12.705 | -4.099 | C | -6.051 | 8.355 | -6.617 |
| N | 6.026 | -8.827 | -9.786 | H | -5.118 | 8.901 | -6.724 |
| C | 8.740 | -9.252 | -10.322 | C | 6.377 | -8.777 | -7.475 |
| C | 6.470 | -8.980 | -11.030 | H | 7.023 | -8.883 | -6.607 |
| C | 6.935 | -8.893 | -8.787 | C | 1.625 | -9.227 | -8.575 |
| C | 8.297 | -9.101 | -9.022 | H | 0.555 | -9.209 | -8.390 |
| C | 7.802 | -9.193 | -11.346 | C | 4.095 | -6.180 | -10.719 |
| H | 5.734 | -8.941 | -11.817 | H | 4.150 | -5.164 | -11.099 |
| H | 8.982 | -9.146 | -8.190 | N | 4.415 | -6.468 | -9.499 |
| H | 8.096 | -9.316 | -12.375 | N | 5.100 | -8.606 | -7.360 |
| H | 9.783 | -9.418 | -10.537 | N | 2.367 | -8.175 | -8.469 |
| N | 3.495 | -8.425 | -11.019 | N | -7.938 | 5.209 | -5.839 |
| C | 2.876 | -8.023 | -13.719 | N | -8.339 | 7.095 | -3.512 |
| C | 3.635 | -7.212 | -11.594 | N | -6.391 | 7.772 | -5.512 |
| C | 3.057 | -9.421 | -11.784 | N | 9.026 | 4.831 | 3.355 |
| C | 2.737 | -9.272 | -13.124 | N | 8.072 | 6.666 | 1.344 |
| C | 3.332 | -6.978 | -12.940 | N | 7.603 | 7.304 | 4.069 |
| H | 2.963 | -10.390 | -11.322 | N | -4.262 | -4.837 | 9.417 |
| H | 2.390 | -10.121 | -13.690 | N | -2.479 | -7.300 | 8.510 |
| H | 3.464 | -5.990 | -13.351 | N | -4.918 | -6.596 | 6.990 |
| H | 2.640 | -7.878 | -14.761 | C | -1.885 | -7.421 | 7.256 |
| N | 3.641 | -10.325 | -8.992 | C | -0.607 | -7.628 | 4.767 |
| C | 2.448 | -12.843 | -9.071 | C | -1.713 | -6.294 | 6.450 |
| C | 2.300 | -10.464 | -8.842 | C | -1.428 | -8.660 | 6.789 |
| C | 4.371 | -11.421 | -9.158 | C | -0.817 | -8.764 | 5.562 |
| C | 3.825 | -12.694 | -9.203 | C | -1.076 | -6.392 | 5.235 |
| C | 1.676 | -11.713 | -8.882 | H | -2.028 | -5.335 | 6.827 |
| H | 5.436 | -11.276 | -9.264 | H | -1.587 | -9.544 | 7.391 |
| H | 4.465 | -13.550 | -9.341 | H | -0.481 | -9.727 | 5.205 |
| H | 0.605 | -11.779 | -8.767 | H | -0.920 | -5.506 | 4.636 |
| H | 1.996 | -13.821 | -9.113 | C | -3.224 | -3.926 | 9.273 |
| C | -1.901 | -7.871 | 9.519 | C | -1.101 | -2.174 | 8.685 |
| H | -0.937 | -8.365 | 9.431 | C | -3.469 | -2.590 | 8.905 |
| C | -5.400 | -4.421 | 9.875 | C | -1.894 | -4.339 | 9.408 |
| H | -5.537 | -3.414 | 10.258 | C | -0.855 | -3.481 | 9.131 |
| C | -5.032 | -7.745 | 6.400 | C | -2.433 | -1.740 | 8.610 |
| H | -5.298 | -7.829 | 5.350 | H | -4.486 | -2.250 | 8.774 |
| C | 7.725 | 7.866 | 1.009 | H | -1.693 | -5.339 | 9.750 |
| H | 7.054 | 8.063 | 0.177 | H | 0.164 | -3.820 | 9.239 |
| C | 7.972 | 7.843 | 5.185 | H | -2.641 | -0.730 | 8.289 |
| H | 7.277 | 8.366 | 5.834 | C | -5.304 | -5.465 | 6.280 |
| C | 10.035 | 4.168 | 2.896 | C | -6.005 | -3.185 | 4.778 |

| | | | |
|---|---|---|---|
| C | -6.423 | -5.475 | 5.430 |
| C | -4.576 | -4.278 | 6.389 |
| C | -4.914 | -3.165 | 5.661 |
| C | -6.766 | -4.361 | 4.701 |
| H | -7.044 | -6.358 | 5.379 |
| H | -3.710 | -4.261 | 7.023 |
| H | -4.330 | -2.262 | 5.755 |
| H | -7.633 | -4.386 | 4.057 |
| C | 7.535 | 5.606 | 0.615 |
| C | 6.504 | 3.480 | -0.924 |
| C | 8.352 | 4.540 | 0.222 |
| C | 6.195 | 5.596 | 0.202 |
| C | 5.687 | 4.549 | -0.529 |
| C | 7.853 | 3.509 | -0.540 |
| H | 9.400 | 4.564 | 0.468 |
| H | 5.543 | 6.404 | 0.494 |
| H | 4.645 | 4.548 | -0.816 |
| H | 8.510 | 2.713 | -0.858 |
| C | 6.292 | 7.482 | 3.646 |
| C | 3.635 | 7.799 | 2.824 |
| C | 5.673 | 8.738 | 3.706 |
| C | 5.565 | 6.389 | 3.165 |
| C | 4.253 | 6.540 | 2.789 |
| C | 4.376 | 8.898 | 3.281 |
| H | 6.241 | 9.593 | 4.047 |
| H | 6.039 | 5.423 | 3.126 |
| H | 3.688 | 5.684 | 2.450 |
| H | 3.917 | 9.875 | 3.300 |
| C | 7.971 | 4.130 | 3.933 |
| C | 5.797 | 2.760 | 5.064 |
| C | 7.499 | 4.471 | 5.205 |
| C | 7.358 | 3.076 | 3.246 |
| C | 6.278 | 2.421 | 3.791 |
| C | 6.443 | 3.790 | 5.764 |
| H | 8.004 | 5.245 | 5.760 |
| H | 7.712 | 2.818 | 2.258 |
| H | 5.792 | 1.632 | 3.237 |
| H | 6.106 | 4.041 | 6.758 |
| C | -6.765 | 4.456 | -5.791 |
| C | -4.458 | 2.847 | -5.783 |
| C | -6.722 | 3.267 | -5.050 |
| C | -5.624 | 4.841 | -6.501 |
| C | -4.500 | 4.044 | -6.511 |
| C | -5.584 | 2.497 | -5.024 |
| H | -7.581 | 2.976 | -4.466 |
| H | -5.654 | 5.745 | -7.089 |
| H | -3.642 | 4.334 | -7.099 |

| | | | |
|---|---|---|---|
| H | -5.558 | 1.598 | -4.427 |
| C | -8.111 | 6.041 | -2.637 |
| C | -7.557 | 3.856 | -0.958 |
| C | -7.246 | 6.177 | -1.540 |
| C | -8.748 | 4.810 | -2.830 |
| C | -8.487 | 3.744 | -2.002 |
| C | -6.957 | 5.104 | -0.734 |
| H | -6.760 | 7.123 | -1.358 |
| H | -9.487 | 4.724 | -3.610 |
| H | -9.003 | 2.805 | -2.146 |
| H | -6.261 | 5.215 | 0.084 |
| C | -5.565 | 7.895 | -4.401 |
| C | -3.853 | 8.085 | -2.192 |
| C | -5.051 | 9.136 | -4.004 |
| C | -5.224 | 6.753 | -3.671 |
| C | -4.366 | 6.846 | -2.602 |
| C | -4.227 | 9.232 | -2.907 |
| H | -5.338 | 10.026 | -4.546 |
| H | -5.613 | 5.798 | -3.986 |
| H | -4.079 | 5.959 | -2.058 |
| H | -3.855 | 10.196 | -2.594 |
| C | 4.615 | -5.411 | -8.624 |
| C | 4.816 | -3.369 | -6.713 |
| C | 5.606 | -5.454 | -7.639 |
| C | 3.777 | -4.286 | -8.683 |
| C | 3.865 | -3.295 | -7.739 |
| C | 5.711 | -4.448 | -6.708 |
| H | 6.310 | -6.270 | -7.651 |
| H | 2.998 | -4.242 | -9.429 |
| H | 3.172 | -2.467 | -7.763 |
| H | 6.486 | -4.480 | -5.956 |
| C | 4.569 | -8.502 | -6.075 |
| C | 3.458 | -8.274 | -3.506 |
| C | 3.448 | -9.257 | -5.713 |
| C | 5.150 | -7.662 | -5.115 |
| C | 4.588 | -7.529 | -3.867 |
| C | 2.917 | -9.161 | -4.449 |
| H | 3.034 | -9.954 | -6.422 |
| H | 6.018 | -7.077 | -5.376 |
| H | 5.024 | -6.850 | -3.148 |
| H | 2.079 | -9.781 | -4.166 |
| C | 1.785 | -6.956 | -8.110 |
| C | 0.662 | -4.498 | -7.347 |
| C | 0.729 | -6.403 | -8.838 |
| C | 2.258 | -6.274 | -6.986 |
| C | 1.685 | -5.086 | -6.594 |
| C | 0.195 | -5.185 | -8.477 |

| | | | |
|---|---|---|---|
| H | 0.361 | -6.920 | -9.714 |
| H | 3.060 | -6.702 | -6.413 |
| H | 2.046 | -4.584 | -5.707 |
| H | -0.594 | -4.750 | -9.072 |
| C | 2.881 | -8.145 | -2.229 |
| C | 2.376 | -8.048 | -1.126 |
| C | 0.130 | -3.242 | -6.987 |
| C | -0.307 | -2.151 | -6.668 |
| C | -7.242 | 2.758 | -0.139 |
| C | -6.952 | 1.821 | 0.585 |
| C | -3.365 | 1.959 | -5.844 |
| C | -2.489 | 1.114 | -5.908 |
| C | -2.958 | 8.153 | -1.109 |
| C | -2.163 | 8.188 | -0.187 |
| C | 0.064 | -7.725 | 3.533 |
| C | 0.660 | -7.799 | 2.473 |
| C | -0.056 | -1.338 | 8.254 |
| C | 0.822 | -0.631 | 7.794 |
| C | -6.280 | -2.064 | 3.977 |
| C | -6.430 | -1.082 | 3.271 |
| C | 2.316 | 7.950 | 2.362 |
| C | 1.184 | 8.075 | 1.928 |
| C | 4.709 | 2.071 | 5.633 |
| C | 3.777 | 1.469 | 6.137 |
| Pt | -1.311 | -0.472 | -6.243 |
| P | 0.615 | 0.631 | -5.600 |
| P | -3.243 | -1.419 | -7.068 |
| Pt | 1.528 | -7.937 | 0.673 |
| P | 3.282 | -9.081 | 1.665 |
| P | -0.480 | -7.156 | -0.193 |
| Pt | -6.651 | 0.380 | 1.936 |
| P | -4.645 | -0.241 | 0.945 |
| P | -8.774 | 0.889 | 2.688 |
| Pt | 2.305 | 0.430 | 6.989 |
| Pt | -0.559 | 8.175 | 0.978 |
| C | 5.990 | 2.423 | -1.701 |
| C | 5.502 | 1.526 | -2.365 |
| C | 4.856 | -2.377 | -5.718 |
| C | 4.845 | -1.500 | -4.873 |
| Pt | 5.079 | -0.010 | -3.581 |
| C | 2.613 | -10.277 | 2.899 |
| H | 1.692 | -10.668 | 2.451 |
| H | 2.312 | -9.680 | 3.766 |
| C | 3.482 | -11.454 | 3.332 |
| H | 4.413 | -11.105 | 3.777 |
| H | 3.726 | -12.074 | 2.468 |
| C | 2.746 | -12.317 | 4.358 |
| H | 1.812 | -12.681 | 3.922 |
| H | 2.492 | -11.707 | 5.228 |
| C | 3.594 | -13.504 | 4.799 |
| H | 4.520 | -13.165 | 5.256 |
| H | 3.841 | -14.135 | 3.950 |
| H | 3.056 | -14.107 | 5.525 |
| C | 4.332 | -10.054 | 0.502 |
| H | 4.381 | -9.461 | -0.417 |
| H | 3.758 | -10.956 | 0.263 |
| C | 5.750 | -10.425 | 0.928 |
| H | 6.329 | -9.518 | 1.112 |
| H | 5.742 | -11.017 | 1.842 |
| C | 6.443 | -11.236 | -0.168 |
| H | 6.458 | -10.654 | -1.094 |
| H | 5.873 | -12.147 | -0.359 |
| C | 7.867 | -11.606 | 0.228 |
| H | 8.463 | -10.714 | 0.405 |
| H | 8.343 | -12.184 | -0.560 |
| H | 7.869 | -12.205 | 1.134 |
| C | 4.394 | -7.920 | 2.571 |
| H | 3.733 | -7.132 | 2.946 |
| H | 5.041 | -7.466 | 1.813 |
| C | 5.227 | -8.447 | 3.736 |
| H | 5.891 | -9.246 | 3.411 |
| H | 4.567 | -8.843 | 4.510 |
| C | 6.073 | -7.323 | 4.337 |
| H | 5.417 | -6.504 | 4.647 |
| H | 6.750 | -6.936 | 3.573 |
| C | 6.885 | -7.809 | 5.532 |
| H | 7.478 | -6.999 | 5.949 |
| H | 7.561 | -8.606 | 5.235 |
| H | 6.231 | -8.193 | 6.311 |
| C | -1.317 | -5.824 | 0.778 |
| H | -0.742 | -4.905 | 0.634 |
| H | -1.186 | -6.120 | 1.821 |
| C | -2.800 | -5.588 | 0.476 |
| H | -2.901 | -4.781 | -0.254 |
| H | -3.251 | -6.482 | 0.043 |
| C | -3.585 | -5.219 | 1.738 |
| H | -3.042 | -4.448 | 2.286 |
| H | -4.551 | -4.803 | 1.445 |
| C | -3.834 | -6.412 | 2.654 |
| H | -2.901 | -6.867 | 2.977 |
| H | -4.427 | -7.166 | 2.140 |
| H | -4.377 | -6.089 | 3.539 |
| C | -0.721 | -6.534 | -1.917 |
| H | 0.020 | -5.743 | -2.067 |

| | | | |
|---|---|---|---|
| H | -1.715 | -6.081 | -1.973 |
| C | -0.569 | -7.582 | -3.014 |
| H | -1.344 | -8.343 | -2.910 |
| H | 0.402 | -8.070 | -2.916 |
| C | -0.687 | -6.938 | -4.395 |
| H | 0.109 | -6.200 | -4.521 |
| H | -1.642 | -6.411 | -4.465 |
| C | -0.598 | -7.965 | -5.517 |
| H | -0.726 | -7.477 | -6.481 |
| H | -1.372 | -8.720 | -5.406 |
| H | 0.373 | -8.456 | -5.505 |
| C | -1.621 | -8.588 | -0.016 |
| H | -2.463 | -8.511 | -0.709 |
| H | -2.002 | -8.539 | 1.007 |
| C | -0.878 | -9.911 | -0.219 |
| H | 0.097 | -9.857 | 0.295 |
| H | -0.679 | -10.059 | -1.282 |
| C | -1.653 | -11.112 | 0.324 |
| H | -1.178 | -12.025 | -0.038 |
| H | -2.673 | -11.096 | -0.068 |
| C | -1.692 | -11.156 | 1.847 |
| H | -0.681 | -11.160 | 2.252 |
| H | -2.197 | -12.059 | 2.181 |
| H | -2.219 | -10.300 | 2.262 |
| C | -3.625 | -0.366 | -8.548 |
| H | -2.644 | 0.078 | -8.768 |
| H | -4.261 | 0.454 | -8.204 |
| C | -4.175 | -0.972 | -9.843 |
| H | -3.815 | -1.993 | -9.973 |
| H | -3.773 | -0.390 | -10.675 |
| C | -5.697 | -0.945 | -9.959 |
| H | -6.151 | -1.522 | -9.153 |
| H | -6.046 | 0.086 | -9.862 |
| C | -6.157 | -1.510 | -11.298 |
| H | -5.835 | -2.543 | -11.411 |
| H | -5.744 | -0.933 | -12.121 |
| H | -7.240 | -1.482 | -11.373 |
| C | -3.160 | -3.173 | -7.629 |
| H | -2.602 | -3.701 | -6.848 |
| H | -2.522 | -3.172 | -8.518 |
| C | -4.466 | -3.912 | -7.906 |
| H | -5.058 | -3.389 | -8.654 |
| H | -5.058 | -3.977 | -6.991 |
| C | -4.185 | -5.329 | -8.409 |
| H | -3.578 | -5.862 | -7.672 |
| H | -3.609 | -5.275 | -9.336 |
| C | -5.476 | -6.097 | -8.663 |

| | | | |
|---|---|---|---|
| H | -5.260 | -7.099 | -9.025 |
| H | -6.083 | -5.591 | -9.410 |
| H | -6.059 | -6.183 | -7.749 |
| C | -4.637 | -1.389 | -5.846 |
| H | -4.594 | -2.348 | -5.319 |
| H | -4.377 | -0.608 | -5.125 |
| C | -6.046 | -1.131 | -6.365 |
| H | -6.335 | -1.898 | -7.082 |
| H | -6.077 | -0.164 | -6.870 |
| C | -7.063 | -1.128 | -5.224 |
| H | -7.112 | -2.124 | -4.779 |
| H | -6.736 | -0.433 | -4.445 |
| C | -8.448 | -0.724 | -5.713 |
| H | -8.801 | -1.414 | -6.475 |
| H | -8.421 | 0.275 | -6.145 |
| H | -9.162 | -0.729 | -4.893 |
| C | 0.493 | 2.443 | -5.282 |
| H | 1.441 | 2.795 | -4.863 |
| H | -0.282 | 2.566 | -4.519 |
| C | 0.108 | 3.249 | -6.518 |
| H | -0.707 | 2.735 | -7.036 |
| H | 0.958 | 3.315 | -7.201 |
| C | -0.351 | 4.659 | -6.150 |
| H | 0.418 | 5.150 | -5.549 |
| H | -1.255 | 4.590 | -5.540 |
| C | -0.632 | 5.498 | -7.391 |
| H | -1.376 | 5.014 | -8.020 |
| H | 0.273 | 5.628 | -7.979 |
| H | -1.001 | 6.482 | -7.112 |
| C | 1.417 | 0.094 | -4.019 |
| H | 1.099 | 0.776 | -3.225 |
| H | 2.516 | 0.178 | -4.125 |
| C | 1.033 | -1.338 | -3.654 |
| H | 1.699 | -1.696 | -2.865 |
| H | 1.172 | -1.982 | -4.525 |
| C | -0.416 | -1.437 | -3.186 |
| H | -1.084 | -1.091 | -4.002 |
| H | -0.570 | -0.779 | -2.326 |
| C | -0.795 | -2.866 | -2.818 |
| H | -0.667 | -3.518 | -3.678 |
| H | -1.834 | -2.920 | -2.501 |
| H | -0.169 | -3.235 | -2.008 |
| C | 1.985 | 0.482 | -6.817 |
| H | 2.656 | 1.338 | -6.699 |
| H | 2.544 | -0.427 | -6.574 |
| C | 1.446 | 0.407 | -8.244 |
| H | 0.810 | -0.481 | -8.332 |

| | | | | | | | |
|---|---|---|---|---|---|---|---|
| H | 0.823 | 1.280 | -8.452 | C | -5.103 | -1.164 | -0.578 |
| C | 2.564 | 0.339 | -9.282 | H | -4.222 | -1.662 | -0.992 |
| H | 3.229 | -0.497 | -9.047 | H | -5.473 | -0.437 | -1.305 |
| H | 3.156 | 1.257 | -9.237 | C | -6.220 | -2.157 | -0.259 |
| C | 2.014 | 0.167 | -10.692 | H | -5.844 | -2.938 | 0.405 |
| H | 1.433 | -0.751 | -10.765 | H | -7.010 | -1.615 | 0.284 |
| H | 2.822 | 0.122 | -11.416 | C | -6.823 | -2.801 | -1.506 |
| H | 1.368 | 1.001 | -10.954 | H | -7.673 | -3.414 | -1.203 |
| C | -8.856 | 2.669 | 3.175 | H | -7.198 | -2.020 | -2.171 |
| H | -8.363 | 2.731 | 4.152 | C | -5.829 | -3.678 | -2.260 |
| H | -8.213 | 3.186 | 2.454 | H | -5.402 | -4.426 | -1.596 |
| C | -10.204 | 3.381 | 3.217 | H | -5.021 | -3.084 | -2.682 |
| H | -10.858 | 2.933 | 3.963 | H | -6.330 | -4.196 | -3.074 |
| H | -10.690 | 3.308 | 2.243 | C | -3.359 | 0.961 | 0.366 |
| C | -10.022 | 4.859 | 3.562 | H | -2.416 | 0.418 | 0.249 |
| H | -9.525 | 4.948 | 4.530 | H | -3.230 | 1.694 | 1.166 |
| H | -9.377 | 5.329 | 2.813 | C | -3.703 | 1.687 | -0.931 |
| C | -11.360 | 5.586 | 3.615 | H | -3.670 | 0.983 | -1.765 |
| H | -12.002 | 5.151 | 4.376 | H | -4.716 | 2.093 | -0.868 |
| H | -11.872 | 5.521 | 2.657 | C | -2.715 | 2.818 | -1.215 |
| H | -11.215 | 6.636 | 3.855 | H | -1.693 | 2.443 | -1.113 |
| C | -9.349 | -0.142 | 4.103 | H | -2.848 | 3.615 | -0.479 |
| H | -9.427 | -1.152 | 3.684 | C | -2.902 | 3.379 | -2.619 |
| H | -8.509 | -0.169 | 4.805 | H | -2.678 | 2.619 | -3.366 |
| C | -10.632 | 0.243 | 4.848 | H | -2.245 | 4.230 | -2.785 |
| H | -11.147 | -0.670 | 5.154 | H | -3.931 | 3.701 | -2.767 |
| H | -11.312 | 0.787 | 4.195 | C | -3.573 | -1.477 | 1.798 |
| C | -10.358 | 1.076 | 6.100 | H | -2.831 | -1.846 | 1.082 |
| H | -9.745 | 0.491 | 6.791 | H | -4.219 | -2.308 | 2.088 |
| H | -9.791 | 1.971 | 5.838 | C | -2.888 | -0.925 | 3.044 |
| C | -11.653 | 1.481 | 6.792 | H | -3.658 | -0.557 | 3.727 |
| H | -12.225 | 0.603 | 7.081 | H | -2.241 | -0.088 | 2.777 |
| H | -12.272 | 2.086 | 6.133 | C | -2.055 | -1.999 | 3.744 |
| H | -11.444 | 2.060 | 7.687 | H | -1.294 | -2.373 | 3.054 |
| C | -9.912 | 0.596 | 1.253 | H | -2.707 | -2.836 | 4.005 |
| H | -9.335 | -0.088 | 0.618 | C | -1.380 | -1.480 | 5.008 |
| H | -9.970 | 1.542 | 0.711 | H | -0.890 | -2.290 | 5.542 |
| C | -11.307 | -0.009 | 1.460 | H | -2.111 | -1.032 | 5.677 |
| H | -11.299 | -0.678 | 2.321 | H | -0.628 | -0.728 | 4.779 |
| H | -11.532 | -0.623 | 0.585 | P | 5.928 | 1.370 | -5.226 |
| C | -12.444 | 1.001 | 1.614 | P | 4.504 | -1.375 | -1.787 |
| H | -12.237 | 1.690 | 2.433 | C | 4.876 | 2.889 | -5.248 |
| H | -13.349 | 0.449 | 1.873 | H | 5.166 | 3.459 | -4.363 |
| C | -12.716 | 1.797 | 0.342 | H | 3.859 | 2.520 | -5.060 |
| H | -11.864 | 2.416 | 0.063 | C | 4.865 | 3.775 | -6.489 |
| H | -13.571 | 2.450 | 0.490 | H | 5.865 | 4.162 | -6.685 |
| H | -12.940 | 1.127 | -0.485 | H | 4.549 | 3.186 | -7.352 |

| | | | |
|---|---|---|---|
| C | 3.906 | 4.959 | -6.334 |
| H | 3.827 | 5.462 | -7.299 |
| H | 2.912 | 4.592 | -6.068 |
| C | 4.367 | 5.974 | -5.294 |
| H | 5.371 | 6.323 | -5.524 |
| H | 4.368 | 5.549 | -4.293 |
| H | 3.703 | 6.835 | -5.294 |
| C | 5.970 | 0.668 | -6.933 |
| H | 4.928 | 0.584 | -7.257 |
| H | 6.336 | -0.356 | -6.796 |
| C | 6.807 | 1.344 | -8.016 |
| H | 7.847 | 1.417 | -7.700 |
| H | 6.445 | 2.351 | -8.221 |
| C | 6.756 | 0.535 | -9.313 |
| H | 5.720 | 0.458 | -9.654 |
| H | 7.116 | -0.479 | -9.121 |
| C | 7.603 | 1.179 | -10.403 |
| H | 7.558 | 0.594 | -11.317 |
| H | 8.643 | 1.245 | -10.093 |
| H | 7.248 | 2.183 | -10.623 |
| H | 8.321 | 1.259 | -5.514 |
| C | 7.685 | 1.807 | -4.816 |
| H | 7.841 | 1.364 | -3.827 |
| C | 8.104 | 3.280 | -4.754 |
| H | 8.892 | 3.373 | -4.003 |
| H | 7.268 | 3.890 | -4.408 |
| C | 8.643 | 3.844 | -6.070 |
| H | 8.705 | 4.930 | -5.978 |
| H | 7.953 | 3.619 | -6.883 |
| C | 10.027 | 3.311 | -6.422 |
| H | 10.743 | 3.561 | -5.643 |
| H | 10.372 | 3.753 | -7.352 |
| H | 10.024 | 2.230 | -6.546 |
| C | 6.100 | -1.507 | -0.876 |
| H | 6.257 | -0.552 | -0.370 |
| H | 6.068 | -2.301 | -0.126 |
| C | 7.236 | -1.753 | -1.870 |
| H | 7.165 | -1.007 | -2.681 |
| H | 7.112 | -2.739 | -2.324 |
| C | 8.621 | -1.671 | -1.231 |
| H | 9.356 | -2.052 | -1.942 |
| H | 8.653 | -2.315 | -0.349 |
| C | 9.014 | -0.252 | -0.841 |
| H | 8.974 | 0.399 | -1.712 |
| H | 10.028 | -0.240 | -0.449 |
| H | 8.349 | 0.148 | -0.078 |
| C | 4.058 | -3.153 | -2.128 |
| H | 5.006 | -3.685 | -2.241 |
| H | 3.584 | -3.155 | -3.113 |
| C | 3.161 | -3.900 | -1.139 |
| H | 2.819 | -4.822 | -1.618 |
| H | 2.271 | -3.306 | -0.921 |
| C | 3.848 | -4.277 | 0.171 |
| H | 4.335 | -3.399 | 0.600 |
| H | 4.622 | -5.020 | -0.027 |
| C | 2.857 | -4.832 | 1.185 |
| H | 3.349 | -5.012 | 2.137 |
| H | 2.427 | -5.786 | 0.830 |
| H | 2.042 | -4.130 | 1.352 |
| H | 2.302 | -1.032 | -0.812 |
| C | 3.322 | -0.841 | -0.469 |
| H | 3.513 | -1.454 | 0.412 |
| C | 3.464 | 0.636 | -0.120 |
| H | 4.506 | 0.862 | 0.109 |
| H | 3.190 | 1.236 | -0.991 |
| C | 2.604 | 1.044 | 1.076 |
| H | 1.550 | 0.862 | 0.856 |
| H | 2.729 | 2.118 | 1.227 |
| C | 3.000 | 0.308 | 2.351 |
| H | 2.636 | -0.716 | 2.344 |
| H | 2.603 | 0.798 | 3.237 |
| H | 4.084 | 0.284 | 2.441 |
| P | 0.183 | 10.147 | 0.046 |
| P | -1.294 | 6.556 | 2.437 |
| P | 1.266 | 2.272 | 7.908 |
| P | 3.436 | -1.533 | 6.500 |
| C | 1.968 | 10.240 | -0.429 |
| H | 2.020 | 10.011 | -1.498 |
| H | 2.452 | 9.415 | 0.104 |
| C | 2.725 | 11.531 | -0.123 |
| H | 2.615 | 11.781 | 0.933 |
| H | 2.330 | 12.358 | -0.712 |
| C | 4.213 | 11.369 | -0.434 |
| H | 4.337 | 11.129 | -1.493 |
| H | 4.609 | 10.528 | 0.146 |
| C | 4.994 | 12.636 | -0.107 |
| H | 6.041 | 12.529 | -0.384 |
| H | 4.937 | 12.862 | 0.955 |
| H | 4.591 | 13.482 | -0.657 |
| C | -0.767 | 10.864 | -1.359 |
| H | -0.933 | 10.035 | -2.056 |
| H | -1.746 | 11.130 | -0.949 |
| C | -0.161 | 12.045 | -2.112 |
| H | 0.784 | 11.750 | -2.570 |

| | | | | | | | |
|---|---|---|---|---|---|---|---|
| H | 0.034 | 12.876 | -1.436 | C | -3.006 | 5.861 | 2.290 |
| C | -1.109 | 12.525 | -3.213 | H | -3.697 | 6.673 | 2.529 |
| H | -1.319 | 11.697 | -3.896 | H | -3.131 | 5.645 | 1.223 |
| H | -2.055 | 12.836 | -2.764 | C | -3.323 | 4.609 | 3.106 |
| C | -0.515 | 13.692 | -3.992 | H | -3.336 | 4.848 | 4.169 |
| H | 0.421 | 13.403 | -4.465 | H | -2.549 | 3.860 | 2.935 |
| H | -0.319 | 14.533 | -3.331 | C | -4.666 | 3.977 | 2.736 |
| H | -1.201 | 14.023 | -4.766 | H | -5.474 | 4.693 | 2.899 |
| C | -0.091 | 11.245 | 1.506 | H | -4.669 | 3.708 | 1.677 |
| H | 0.735 | 11.051 | 2.197 | C | -4.911 | 2.728 | 3.578 |
| H | -1.004 | 10.848 | 1.980 | H | -5.110 | 2.993 | 4.613 |
| C | -0.265 | 12.747 | 1.289 | H | -4.038 | 2.077 | 3.554 |
| H | 0.624 | 13.171 | 0.822 | H | -5.774 | 2.160 | 3.194 |
| H | -1.117 | 12.931 | 0.634 | C | 1.512 | 2.138 | 9.731 |
| C | -0.509 | 13.463 | 2.619 | H | 2.497 | 2.570 | 9.938 |
| H | 0.335 | 13.284 | 3.289 | H | 1.575 | 1.065 | 9.945 |
| H | -1.402 | 13.051 | 3.095 | C | 0.462 | 2.753 | 10.654 |
| C | -0.687 | 14.963 | 2.420 | H | 0.363 | 3.823 | 10.472 |
| H | 0.200 | 15.400 | 1.968 | H | -0.506 | 2.283 | 10.473 |
| H | -0.860 | 15.453 | 3.373 | C | 0.845 | 2.540 | 12.120 |
| H | -1.538 | 15.166 | 1.774 | H | 1.814 | 3.004 | 12.314 |
| C | -1.189 | 7.463 | 4.045 | H | 0.945 | 1.470 | 12.316 |
| H | -0.124 | 7.493 | 4.296 | C | -0.194 | 3.134 | 13.062 |
| H | -1.483 | 8.489 | 3.787 | H | -0.292 | 4.205 | 12.901 |
| C | -2.004 | 7.005 | 5.250 | H | 0.096 | 2.972 | 14.095 |
| H | -1.766 | 5.967 | 5.486 | H | -1.167 | 2.673 | 12.904 |
| H | -3.070 | 7.072 | 5.023 | C | -0.552 | 2.229 | 7.578 |
| C | -1.706 | 7.860 | 6.486 | H | -0.645 | 1.887 | 6.540 |
| H | -0.633 | 7.839 | 6.689 | H | -0.932 | 1.419 | 8.207 |
| H | -2.213 | 7.412 | 7.344 | C | -1.398 | 3.484 | 7.768 |
| C | -2.168 | 9.306 | 6.350 | H | -1.021 | 4.292 | 7.139 |
| H | -1.596 | 9.838 | 5.593 | H | -1.362 | 3.816 | 8.805 |
| H | -2.035 | 9.828 | 7.294 | C | -2.854 | 3.194 | 7.397 |
| H | -3.222 | 9.349 | 6.086 | H | -2.912 | 2.919 | 6.340 |
| C | -0.195 | 5.068 | 2.534 | H | -3.205 | 2.338 | 7.978 |
| H | 0.772 | 5.389 | 2.137 | C | -3.766 | 4.383 | 7.671 |
| H | -0.609 | 4.350 | 1.821 | H | -3.457 | 5.252 | 7.095 |
| C | 0.025 | 4.418 | 3.902 | H | -3.741 | 4.647 | 8.725 |
| H | 0.665 | 5.075 | 4.495 | H | -4.794 | 4.145 | 7.408 |
| H | -0.918 | 4.300 | 4.433 | C | 1.875 | 3.950 | 7.430 |
| C | 0.685 | 3.042 | 3.775 | H | 2.952 | 3.827 | 7.272 |
| H | 1.065 | 2.724 | 4.751 | H | 1.439 | 4.172 | 6.452 |
| H | 1.541 | 3.113 | 3.102 | C | 1.635 | 5.120 | 8.380 |
| C | -0.282 | 1.976 | 3.272 | H | 2.146 | 4.943 | 9.327 |
| H | -1.151 | 1.918 | 3.925 | H | 0.572 | 5.240 | 8.586 |
| H | 0.205 | 1.005 | 3.267 | C | 2.165 | 6.420 | 7.773 |
| H | -0.620 | 2.189 | 2.259 | H | 3.233 | 6.314 | 7.565 |

| | | | |
|---|---|---|---|
| H | 1.664 | 6.603 | 6.818 |
| C | 1.940 | 7.605 | 8.704 |
| H | 2.307 | 8.523 | 8.252 |
| H | 2.462 | 7.456 | 9.645 |
| H | 0.881 | 7.729 | 8.919 |
| C | 4.761 | -1.567 | 7.772 |
| H | 5.579 | -0.936 | 7.416 |
| H | 5.139 | -2.582 | 7.916 |
| C | 4.212 | -0.991 | 9.079 |
| H | 3.395 | -1.617 | 9.448 |
| H | 3.802 | 0.016 | 8.868 |
| C | 5.280 | -0.871 | 10.162 |
| H | 6.106 | -0.260 | 9.791 |
| H | 5.677 | -1.862 | 10.392 |
| C | 4.717 | -0.245 | 11.433 |
| H | 3.897 | -0.843 | 11.825 |
| H | 4.346 | 0.758 | 11.234 |
| H | 5.486 | -0.181 | 12.197 |
| C | 4.302 | -1.753 | 4.883 |
| H | 4.511 | -0.740 | 4.529 |
| H | 3.573 | -2.197 | 4.199 |
| C | 5.599 | -2.560 | 4.874 |
| H | 5.454 | -3.541 | 5.324 |
| H | 6.361 | -2.038 | 5.455 |
| C | 6.107 | -2.743 | 3.444 |
| H | 6.225 | -1.762 | 2.975 |
| H | 5.364 | -3.300 | 2.868 |
| C | 7.436 | -3.486 | 3.409 |
| H | 7.772 | -3.628 | 2.384 |
| H | 7.339 | -4.463 | 3.876 |
| H | 8.200 | -2.929 | 3.945 |
| C | 2.478 | -3.088 | 6.775 |
| H | 1.530 | -2.956 | 6.243 |
| H | 2.240 | -3.089 | 7.841 |
| C | 3.094 | -4.427 | 6.383 |
| H | 3.234 | -4.470 | 5.301 |
| H | 4.066 | -4.559 | 6.862 |
| C | 2.173 | -5.573 | 6.809 |
| H | 1.159 | -5.374 | 6.452 |
| H | 2.137 | -5.615 | 7.900 |
| C | 2.638 | -6.918 | 6.267 |
| H | 2.602 | -6.923 | 5.179 |
| H | 1.993 | -7.716 | 6.628 |
| H | 3.656 | -7.129 | 6.586 |
